# Evidence of sociodemographic heterogeneity across the HIV treatment cascade and progress towards 90‐90‐90 in sub‐Saharan Africa – a systematic review and meta‐analysis

**DOI:** 10.1002/jia2.25470

**Published:** 2020-03-09

**Authors:** Dylan Green, Diana M Tordoff, Brenda Kharono, Adam Akullian, Anna Bershteyn, Michelle Morrison, Geoff Garnett, Ann Duerr, Paul K Drain

**Affiliations:** ^1^ Department of Epidemiology University of Washington Seattle WA USA; ^2^ Strategic Analysis, Research & Training (START) Center University of Washington Seattle WA USA; ^3^ Department of Global Health University of Washington Seattle WA USA; ^4^ Institute for Disease Modeling (IDM) Bellevue WA USA; ^5^ Bill and Melinda Gates Foundation Seattle WA USA; ^6^ Vaccine and Infectious Disease Public Health Science Divisions Fred Hutchinson Cancer Resesarch Center HIV Vaccine Trials Network Seattle WA USA; ^7^ Division of Allergy and Infectious Diseases Department of Medicine School of Medicine University of Washington Seattle WA USA

**Keywords:** 90‐90‐90, HIV testing, antiretroviral treatment, cascade, sub‐Saharan Africa, sustained virologic response

## Abstract

**Introduction:**

Heterogeneity of sociodemographics and risk behaviours across the HIV treatment cascade could influence the public health impact of universal ART in sub‐Saharan Africa if those not virologically suppressed are more likely to be part of a risk group contributing to onward infections. Sociodemographic and risk heterogeneity across the treatment cascade has not yet been comprehensively described or quantified and we seek to systematically review and synthesize research on this topic among adults in Africa.

**Methods:**

We conducted a systematic review of peer‐reviewed literature in Embase and MEDLINE databases as well as grey literature sources published in English between 2014 and 2018. We included studies that included people living with HIV (PLHIV) aged ≥15 years, and reported a 90‐90‐90 outcome: awareness of HIV‐positive status, ART use among those diagnosed or viral suppression among those on ART. We summarized measures of association between sociodemographics, within each outcome, and as a composite measure of population‐wide viral suppression.

**Results and discussion:**

From 3533 screened titles, we extracted data from 92 studies (50 peer‐reviewed, 42 grey sources). Of included studies, 32 reported on awareness, 53 on ART use, 32 on viral suppression and 23 on population‐wide viral suppression. The majority of studies were conducted in South Africa, Uganda, and Malawi and reported data for age and gender. When stratified, PLHIV ages 15 to 24 years had lower median achievement of the treatment cascade (60‐49‐81), as compared to PLHIV ≥25 years (70‐63‐91). Men also had lower median achievement of the treatment cascade (66‐72‐85), compared to women (79‐76‐89). For population‐wide viral suppression, women aged ≥45 years had achieved the 73% target, while the lowest medians were among 15‐ to 24‐year‐old men (37%) and women (49%).

**Conclusions:**

Considerable heterogeneity exists by age and gender for achieving the HIV 90‐90‐90 treatment goals. These results may inform delivery of HIV testing and treatment in sub‐Saharan Africa, as targeting youth and men could be a strategic way to maximize the population‐level impact of ART.

## 1 Introduction

In 2014, the Joint United Nations Programme on HIV/AIDS (UNAIDS) introduced the 90‐90‐90 goals as targets for achieving viral load suppression among people living with HIV (PLHIV) by 2020 [1]. The 90‐90‐90 goals were introduced alongside universal testing and treatment (UTT), regardless of CD4 count, with the hope of ending the HIV epidemic by 2030 [2]. Despite encouraging trends in antiretroviral therapy (ART) coverage and levels of viral load suppression, HIV incidence has not declined as much as predicted in HIV simulations, and remains high in young women in sub‐Saharan Africa and key populations [3]. Given the mixed results from recent cluster randomized trials of the population‐level effect of UTT on incidence declines and observed ongoing HIV incidence across populations, the UTT strategy and 90‐90‐90 goals may not be reaching all PLHIV [4, 5, 6].

The rationale for UTT, that it will reduce population‐level HIV incidence, has relied primarily on results of mathematical models, which vary in their complexities and assumptions [7, 8, 9, 10, 11, 12]. Empirical data from cluster randomized trials of UTT have generated lower estimates of the population‐level HIV incidence impact of UTT [4, 5, 6]. While there are several potential explanations for these discrepancies, one potential explanation is heterogeneity in viral suppression across sociodemographics and risk behaviours. Models to date have not parameterized the treatment cascade as a function of risk based on empirical observations, despite some evidence that PLHIV who are not receiving ART or achieving viral load suppression are younger, male, highly mobile, and exhibit more risky sexual behaviour, and are thus more likely to transmit HIV to others [13].

Assessing the association of diagnosis, ART use and viral suppression with risk behaviour and sociodemographic factors will allow for a better understanding of the population‐level effectiveness of UTT in reducing HIV incidence, and provide a focus for improving programmes. We conducted a systematic review to summarize and quantify the heterogeneity in sociodemographics and behavioural risk factors across the HIV treatment cascade among adult PLHIV in sub‐Saharan Africa.

## 2 Methods

### 2.1 Search strategy and selection criteria

We conducted a systematic review following the PRISMA guidelines and registered the protocol details on the International Prospective Register of Systematic Reviews (PROSPERO #CRD42018089505), and published the protocol in a peer‐reviewed publication [14]. We searched Embase and MEDLINE databases for English‐language manuscripts published from 1 January 2014 to 6 August 2018. We constructed search terms for each of the four outcomes of interest: awareness of HIV‐positive status, ART use among those aware of their status, viral suppression among those on ART and a composite of viral suppression among all PLHIV (File S1). We refer to these outcomes as *Awareness of HIV‐Positive Status* (First 90), *ART Use* (Second 90), *Viral Suppression* (Third 90) and *Population‐wide Viral Suppression.*

Three authors (BK, DG and DMT) screened titles, abstracts and full‐text publications independently and in duplicate using Excel and Covidence software [15]. Studies were excluded if reviewers were in concurrence, with disputes at the full‐text review stage resolved in consultation with a third reviewer.

In addition to peer‐reviewed sources, we searched pre‐specified grey literature sources using the same inclusion criteria, which included: WHO reports, UNAIDS reports, Médecins Sans Frontières reports, Integrated Bio‐Behavioral Surveys, the Analyzing Longitudinal Population‐based HIV/AIDS data on Africa Network, the National Technical Reports Library, Population‐based HIV Impact Assessments (PHIA), AIDS Indicator Surveys and Demographic and Health Surveys, other similar national HIV surveys (e.g. the South African National HIV Prevalence, Incidence and Behavior Survey), International AIDS Society Conference abstracts, AIDS Conference abstracts, and Conference on Retroviruses and Opportunistic Infections abstracts [16, 17, 18, 19, 20, 21, 22, 23, 24].

Study inclusion criteria were: study includes data only on PLHIV or disaggregates participant data by HIV serostatus, study set in sub‐Saharan Africa, study participants aged 15 or older, original non‐qualitative research, 50% or more of the study period fell between 1 January 2014 or later and reports data on at least one outcome of interest. The rationale for choosing this cutoff date is to omit pre‐UTT studies while aiming to capture potential studies of UTT which later informed policy.

### 2.2 Primary exposure measures

The exposures of interest were selected *a priori* and included the following: age, gender, geography, urban/rural residence, occupation, distance from facility, relationship status, age at sexual debut, recency of infection, CD4 count, education, income or wealth, migration status, mobility (i.e. time spent away from residence), mental health, identification with a key or priority population group (pregnant women, discordant couples, people who inject drugs, transactional/commercial sex workers, gay, bisexual and other men who have sex with men, and transgender individuals) and sexual risk behaviours (number of concurrent partners, condom‐use, age‐discordant relationships).

### 2.3 Primary outcome measures

For *Awareness of HIV‐Positive Status* (First 90), we included studies that featured data on persons previously aware of their status and those previously unaware of their status, including those newly diagnosed. The implicit denominator or sample population for estimates of the First 90 is all PLHIV. For *ART Use* (Second 90) we included studies comparing persons on ART to those not on ART, including studies investigating linkage to and initiation of ART, but not linkage to care, where the endpoint may not have been exclusive to people on ART. The denominator for *ART Use* is PLHIV previously aware of their status. For *Viral Suppression* (Third 90) we included studies regardless of the viral suppression cutoff used in the study, where the denominator is PLHIV on ART. *Population‐wide Viral Suppression* is defined similarly to *Viral Suppression*, but with a denominator of all PLHIV.

### 2.4 Data abstraction and analysis

Two authors (DG and DMT) used a standard data abstraction form to independently extract data, and cross‐check abstracted data for accuracy. We abstracted study metadata including titles, author, publication year, study period, geography, study design and sampling method. We also abstracted data on study participant characteristics as well as demographic and behavioural strata. We abstracted count data of the exposures of interest by outcome and unadjusted measures of association across exposure levels including odds ratios, risk ratios, prevalence ratios, risk differences and hazard ratios. We have not estimated a pooled measure of association but have aggregated unweighted prevalence of achievement of each treatment cascade step by age and gender. Our rationale for this technical approach is that while we do not believe there is an underlying natural causal relationship between sociodemographic and risk behaviour factors across all adults in Africa, we do believe there may be statistical trends or patterns which could most easily be expressed quantitatively with reserved interpretation. We include both measures of association and aggregate count data to highlight study settings where statistically significant associations have been found, as well as qualitative trends across studies. Finally, we summarize the risk of bias within included studies using an adapted risk of bias assessment across standard domains of confounding, selection, misclassification and missing data [25].

## 3 Results

### 3.1 Study characteristics

**Figure 1:**
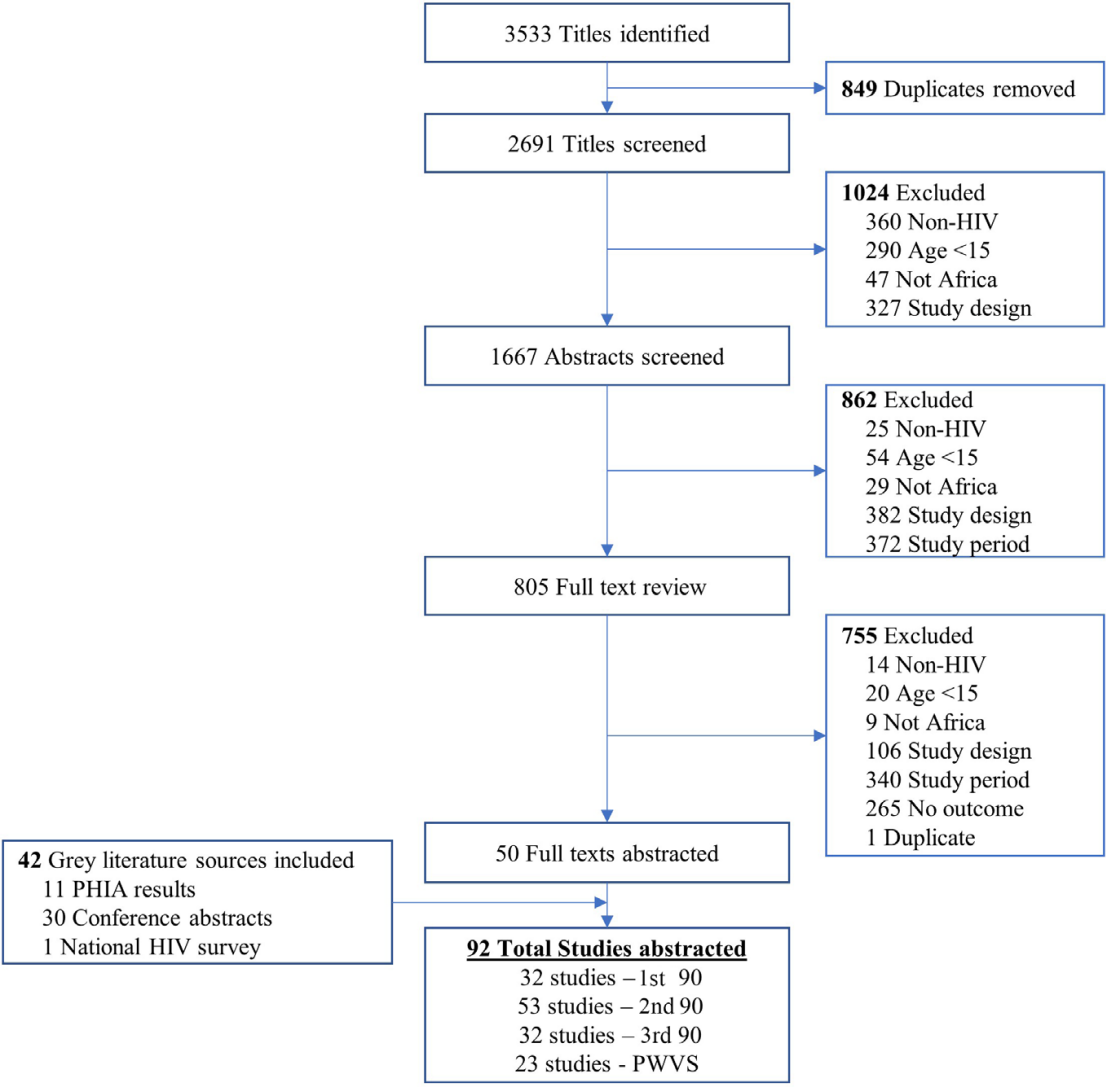
Flow diagram of included studies. PHIA, Population‐based HIV impact assessment.

Overall, our Embase and MEDLINE searches yielded 3533 titles and abstracts for screening (Figure 1). After title and abstract screening, 805 peer‐reviewed sources remained for full text review. Ultimately, 50 studies from the peer‐reviewed literature met inclusion criteria, with an additional 42 studies from grey literature for a total of 92 studies that had data abstracted. The most common reason for exclusion was inappropriate study design (31% of all excluded studies). Note that while 92 studies were included in the review, fewer studies are included in each sub‐analysis.

**Figure 2:**
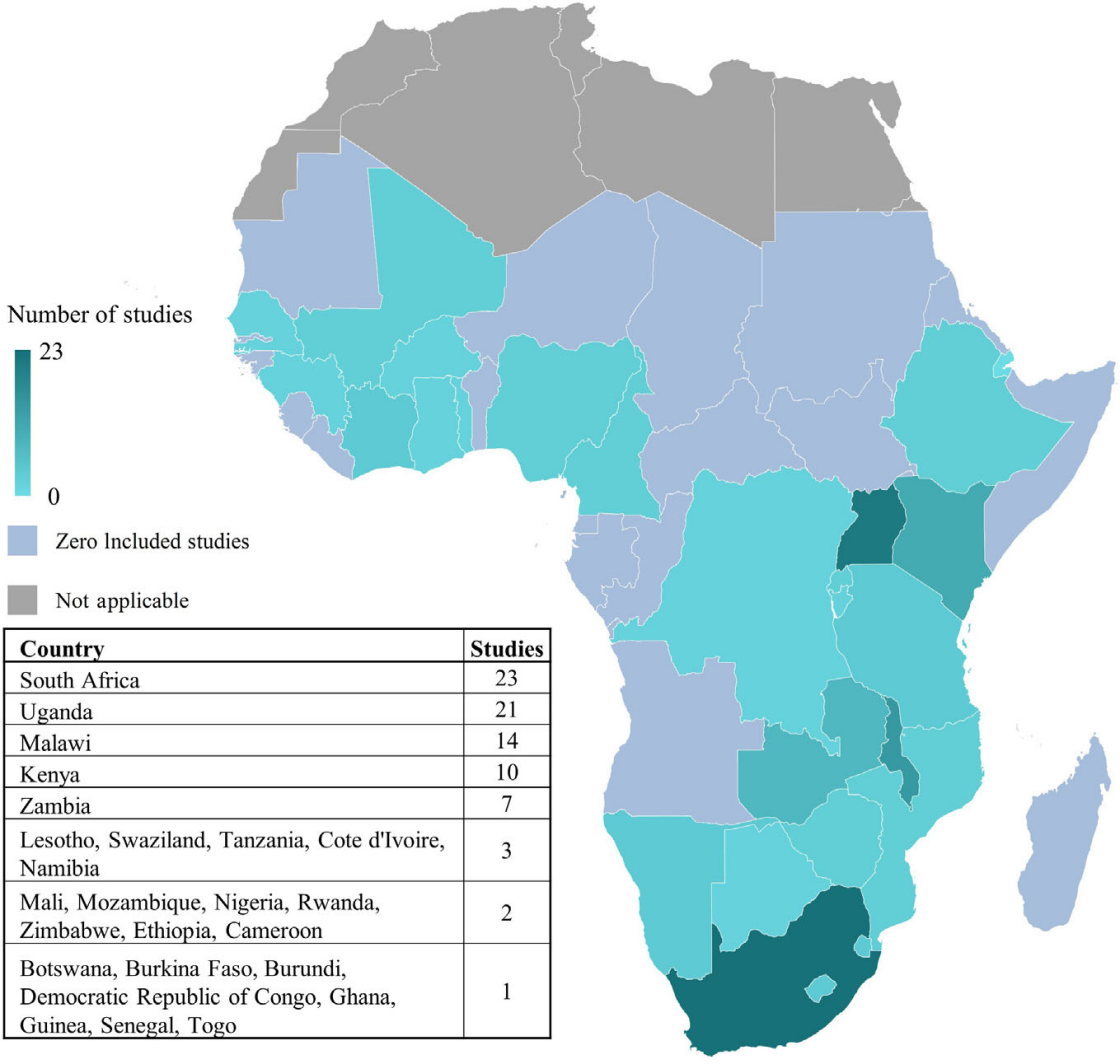
Number of studies by country. Blue gradation reflects number of included studies set in each country, pink colour represents sub‐Sahara African countries with no included data and grey reflects non‐sub‐Saharan countries.

Included studies were most commonly conducted in South Africa (23 studies), Uganda (21 studies) and Malawi (14) (Figure 2). *ART Use* was the most commonly reported outcome (53 studies), followed by *Awareness of HIV‐Positive Status* and *Viral Suppression* (32 studies each), and *Population‐wide Viral Suppression* was the least commonly reported outcome (23 studies). Cross‐sectional study design was most common in included studies (43 studies) followed by cohort studies (33 studies), randomized trials (15 studies) and one case‐control study. Studies primarily used either clinic or facility‐based sampling methods (45 studies), followed by population‐based sampling (21 studies), community‐based sampling (16 studies), respondent driven sampling (6 studies) and venue‐based sampling (4 studies).

A total of nine viral load cutoffs were used in defining the *Viral Suppression* and *Population‐wide Viral Suppression*, with under 1000 copies/mL used most commonly (63%). A complete table of included studies and their characteristics is listed in File S2.

Overall, 58% of included peer‐reviewed studies and population‐based surveys were classified as low risk of bias, while 40% were classified as having some concerns, and 2% were rated as high risk of bias. Bias concerns were primarily related to confounding and selection, with 58% of studies having some confounding concerns, one third of studies having some selection concerns, and another one third having high risk of bias related to selection. Ninety‐five percent and 82% of studies had low risk of bias related to classification or missing data respectively. Each study's risk of bias by domain can be found in File S3.

### 3.2 Target population and risk factor measurement

The majority of included studies targeted general adult populations with their sampling design (53 studies). The remainder of studies targeted specific populations, often groups which are defined by UNAIDS as key or vulnerable populations. The most commonly targeted sub‐populations included pregnant women (12 studies), female sex workers (8 studies), men who have sex with men (7 studies) and youth (6 studies). Only one study included each of the following populations: transgender women, fisherfolk, incarcerated persons and psychotic patients.

**Figure 3:**
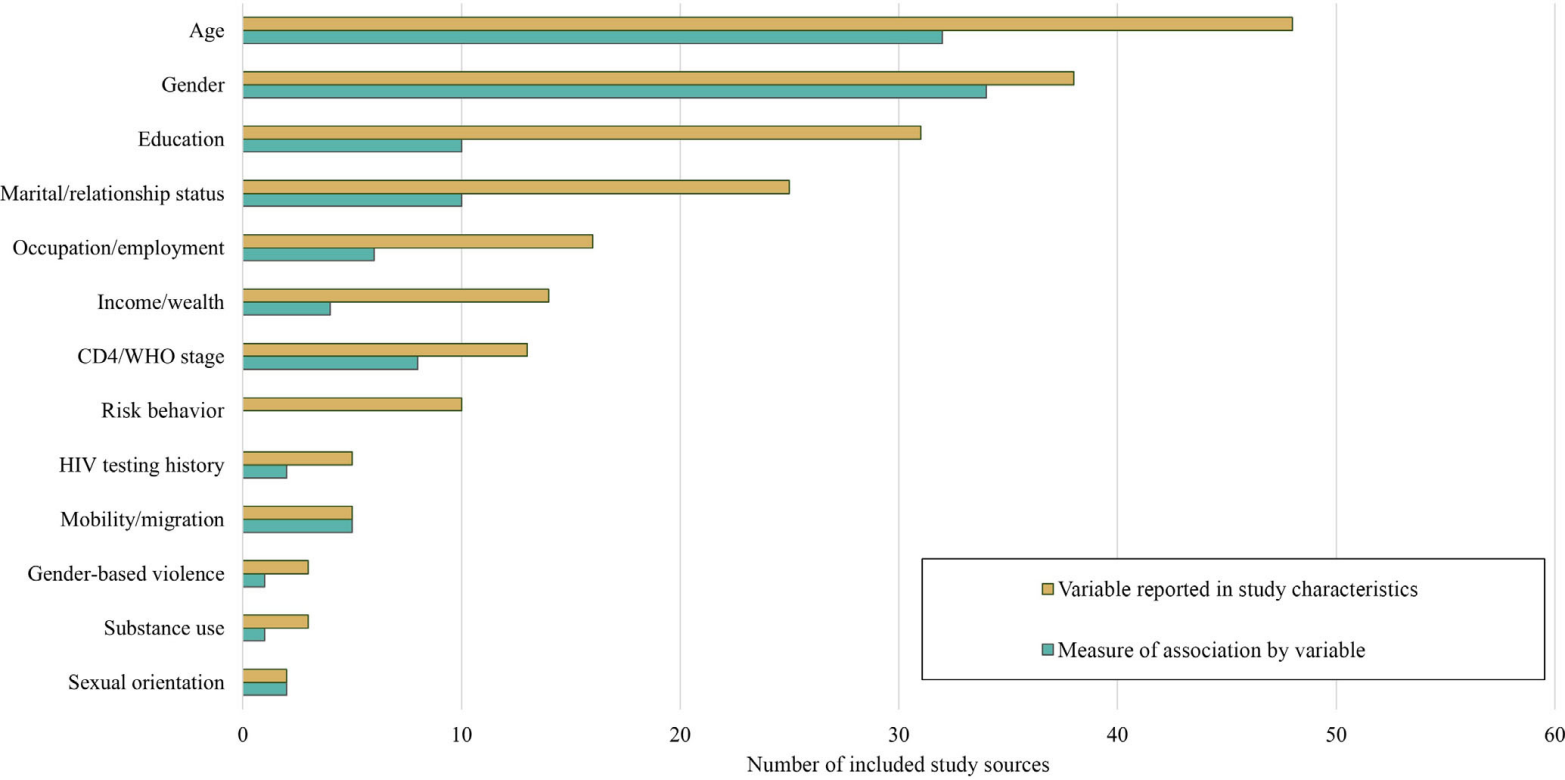
Number of studies which reported each variable and reported a measure of association by each variable. Variable Reported in Study Characteristics reflects the number of included studies which reported the accompanying sociodemographic or risk behaviour data. Measure of Association by Variable represents the number of studies where the sociodemographic or risk behaviour data were analysed by an outcome of interest, that is, an odds ratio or disaggregated count data.

Age was the most commonly documented stratification variable, followed by gender, education, and marital/relationship status in 48, 38, 31 and 25 studies respectively (Figure 3). While each of these strata were frequently reported on in included studies, far fewer studies calculated measures of association between *Awareness of HIV‐Positive Status*, *ART Use*, *Viral Suppression*, or *Population‐wide Viral Suppression* by demographic strata. Ten studies reported sexual risk behaviour such as condom use, transactional sex or concurrent partnerships, but no included study presented a measure of association between the outcomes of interest and sexual risk behaviours. Information on sexual orientation, substance use and gender‐based violence was restricted to studies focused on key populations.

### 3.3 Awareness of HIV‐positive status

We identified 32 studies which met search criteria for the outcome of *Awareness of HIV‐Positive Status*, 29 studies reported only count data, while three studies – located in Botswana, South Africa and Malawi – produced measures of association across strata of interest (Table 1). In Botswana, men had a prevalence ratio of 0·90 for *Awareness of HIV‐Positive Status* relative to women (95% CI 0.87 to 0.94) [26]. Groups aged 16 to 19 had prevalence ratio of 0.74 for *Awareness* relative to those aged 60 and up (95% CI 0.60 to 0.92). Persons who reported spending three or more months away from their community in the past year had a prevalence ratio for *Awareness of HIV‐Positive Status* relative to those spending no time away of 0·83 (95% CI 0.71 to 0.97). High income, less educated, and single groups were all less likely to be aware of their HIV status relative to low income, secondary educated and married persons respectively [26]. In South Africa, women were found to have more than two‐fold greater odds of *Awareness of HIV‐Positive Status* relative to men (95% CI 1.75 to 2.57) [27]. Furthermore, those aged 15 to 19 years had 0.16‐fold lower odds of *Awareness of HIV‐Positive Status* relative to those aged 45 to 49 years (95% CI 0.09 to 0.29). Contrary to the Botswana study, these results found married persons to be more likely than single persons to be aware of their status and found no statistically significant association across education or mobility. The third study was conducted exclusively among female sex workers in Malawi, and investigated associations between substance use and *Awareness of HIV‐Positive Status*, finding those with alcohol dependency had 2.4‐fold greater prevalence of being unaware of their HIV‐positive status compared to non‐harmful use (95% CI 1.0 to 5.6), but found no associations for other levels of alcohol consumption or marijuana use [28].

**Table 1:** Measures of association between selected sociodemographic strata and outcomes for studies presenting measures of association

Strata	Comparison	Awareness of HIV‐positive status	ART use	Viral suppression	Population‐wide viral suppression
Author (publication year) [Reference]: M. Petersen (2017) | Countries: Kenya, Uganda | Study population: General population | n: 77,774 | Measure of association: Risk difference					
Gender	Men versus women				**−2.6 (−0.7 to −4.4)**
Age	15 to 24 versus 44+				**−9.2 (−3.6 to −14.9)**
Relationship status	Never married versus married				**−5.9 (−2.7 to −9.1)**
Education	Secondary versus less than primary				1.8 (−8.5 to 12.1)
Occupation	Unemployed versus formal employment				**−**3.5 (−17 to 10)
Wealth	Richest quintile versus poorest quintile				0.9 (−3.6 to 5.3)
Mobility	1+ month away versus <1 month away				**−**2.6 (−5.9 to 0.6)
Author (publication year) [Reference]: T. Gaolathe (2016) | Country: Botswana | Study population: General population | n: 3596 | Measure of association: Prevalence ratio					
Gender	Men versus women	**0.90 (0.87 to 0.94)**	**1.04 (1.01 to 1.07)**	0.99 (0.97 to 1.01)	**0.92 (0.87 to 0.98)**
Age	16 to 19 versus 60+	**0.74 (0.60 to 0.92)**	**0.8 (0.66 to 0.97)**	**0.70 (0.56 to 0.87)**	**0.41 (0.29 to 0.59)**
Relationship status	Single/never married versus married	**0.92 (0.89 to 0.95)**	**0.93 (0.89 to 0.96)**	**0.97 (0.95 to 0.98)**	**0.83 (0.78 to 0.87)**
Education	More than secondary versus non‐formal	**0.88 (0.83 to 0.94)**	**0.95 (0.90 to 1.00)**	**0.97 (0.94 to 1.00)**	**0.81 (0.73 to 0.89)**
Occupation	Employed versus unemployed	**1.05 (1.01 to 1.09)**	0.99 (0.96 to 1.01)	1.00 (0.98 to 1.01)	1.03 (0.96 to 1.09)
Income	$477+ per month versus none	**0.90 (0.83 to 0.97)**	1.06 (0.99 to 1.13)	0.94 (0.88 to 1.01)	0.88 (0.75 to 1.03)
Mobility	3+ months away versus no time away	**0.83 (0.71 to 0.97)**	0.98 (0.88 to 1.09)	0.96 (0.90 to 1.03)	**0.78 (0.64 to 0.95)**
Author (publication year) [Reference]: A. Grobler (2017) | Country: South Africa | Study population: General population | n: 9812 | Measure of association: Odds ratio					
Gender	Women versus men	**2.12 (1.75 to 2.57)**	1.17 (0.86 to 1.58)	1.6 (0.92 to 2.77)	**2.11 (1.69 to 2.63)**
Age	15 to 19 versus 45 to 49	**0.16 (0.09 to 0.29)**	0.82 (0.27 to 2.51)	0.27 (0.07 to 1.13)	**0.22 (0.13 to 0.38)**
relationship status	Married versus single	**1.51 (1.12 to 2.04)**	1.09 (0.81 to 1.48)	1.13 (0.56 to 2.27)	**1.50 (1.08 to 2.09)**
Education	Secondary versus primary	0.84 (0.57 to 1.22)	0.82 (0.48 to 1.39)	1.35 (0.67 to 2.72)	1.05 (0.74 to 1.50)
Mobility	<1 month away versus 1+ month away	1.00 (0.74 to 1.36)	0.95 (0.66 to 1.38)	**1.81 (1.13 to 2.91)**	1.29 (0.96 to 1.73)
Author (publication year) [Reference]: KE. Lancaster (2016) | Country: Malawi | Study population: Female sex workers | n: 138 | Measure of association: Prevalence ratio					
Alcohol Use	Dependence versus non‐harmful use	**0.42 (0.18 to 1.0)**			
Marijuana Use	Current use versus no current use	0.91 (0.42 to 2.0)			
Author (publication year) [Reference]: S. Boyer (2016) | Country: South Africa | Study population: General population | n: 514 | Measure of association: Hazard ratio					
Gender	Women versus men		1.0 (0.8 to 1.4)		
Age	50+ versus 16 to 29		0.9 (0.6 to 1.3)		
Relationship status	No regular partner versus has regular partner		**1.3 (1.0 to 1.8)**		
Education	Secondary versus less than secondary		**1.3 (1.0 to 1.7)**		
Occupation	Unemployed versus employed		1.0 (0.7 to 1.4)		
Wealth	High wealth versus low wealth		0.7 (0.5 to 1.1)		
Author (publication year) [Reference]: ME. Charurat (2015) | Country: Nigeria | Study population: Men who have sex with men | n: 186 | Measure of association: Odds ratio					
Age	31+ versus 16 to 24		2.25 (0.54 to 9.34)		
Relationship status	Single/never married versus married or cohabitating		2.58 (0.82 to 8.01)		
Education	More than secondary versus less than primary		1.22 (0.38 to 3.92)		
Occupation	Working versus not working		0.63 (0.20 to 2.00)		
Author (publication year) [Reference]: CB. Holmes (2018) | Country: Zambia | Study population: General population | n: 165,464 | Measure of association: Hazard ratio					
Gender	Men versus women		**0.54 (0.31 to 0.93)**		
Author (publication year) [Reference]: Y. Zhang (2018) | Countries: Kenya, Malawi, South Africa | Study population: Men who have sex with men & transgender women | n: 63 | Measure of association: Odds ratio					
Age	26 to 44 versus 18 to 25		**3.23 (1.71 to 6.1)**		
Gender	Transgender women versus men		1.25 (0.65 to 2.4)		
Mobility	Immigrant versus non‐immigrant		3.05 (0.83 to 11.3)		
Author (publication year) [Reference]: D. Kerrigan (2017) | Country: Tanzania | Study population: Female sex workers | n: 496 | Measure of association: Odds ratio					
Age	30+ versus <30			**3.89 (1.32 to 11.45)**	
Relationship status	Ever married versus never married			1.96 (0.67 to 5.6)	
Education	Less than secondary versus secondary or higher			0.55 (0.18 to 1.65)	
Gender‐based violence	Ever versus never			0.59 (0.20 to 1.79)	
Substance use	Ever versus never			0.56 (0.21 to 1.52)	
Author (publication year) [Reference]: MB. Chagomerana (2018) | Country: Malawi | Study population: Pregnant women | n: 299 | Measure of association: Odds ratio					
Education	Secondary or greater versus primary or less			0.82 (0.4 to 1.64)	
relationship status	Single versus married			1.82 (0.35 to 9.38)	
Author (publication year) [Reference]: B. Hansoti (2018) | Country: South Africa | Study population: General population | n: 2100 | Measure of association: Prevalence ratio					
Gender	Women versus men			1.14 (0.94 to 1.37)	
Author (publication year) [Reference]: M. Nsumba (2017) | Country: Uganda | Study population: General population | n: 5867 | Measure of association: Odds ratio					
Gender	Men versus women			1.04 (0.62 to 1.72)	
Age	44+ versus <44			**0.27 (0.13 to 0.54)**	

Bolded values are statistically significant at a 0.05 level.

### 3.4 ART use

We included 53 studies that reported data on *ART Use*, with six studies that produced statistical measures of association across demographic strata. Men were found to be more likely than women to use ART in Botswana while they were less likely to be on ART in Zambia; and no statistically significant association by gender was found in two South African studies [26, 27, 29, 30]. Two studies found older age groups to be significantly more likely to be on ART compared to younger age groups [26, 31], while two studies failed to find any association between age and *ART Use* [30, 32]. One study did not investigate gender as it was conducted exclusively among men [32], and a small study of men who have sex with men (MSM) and transgender women failed to find an association of *ART Use* across those gender identities nor did it find an association by relationship status [31]. However, in the general population, two studies found married groups to be more likely than single and never married groups to be on ART [25, 27], and a third study found those reporting having a regular partner were more likely to use ART than those without a regular partner [30]. Finally, two studies failed to find an association between mobility and *ART Use* [25, 27], and the study of MSM and transgender women found no association between immigrants and non‐immigrants [31].

### 3.5 Viral suppression

We included 32 studies that reported data on *Viral Suppression*, with six that reported measures of association. In Uganda, groups under 44 years old had 0.27‐fold lower odds of *Viral Suppression* compared to older groups (95% CI 0.13 to 0.54) [33]. In Botswana persons aged 16 to 19 had 0.7‐fold lower prevalence of suppression compared to those 60 and up (95% CI 0.56 to 0.87) [26]. In a Tanzanian study of female sex workers, women aged 30 and up had 3.89‐fold greater odds of *Viral Suppression* compared to those under 30 (95% CI 1.32 to 11.45) [34]. Three studies failed to detect an association between gender and *Viral Suppression* [25, 27, 33], and two others did not investigate an effect of gender as they were only conducted among women [34, 35]. Two studies found no association between mobility or migration status on *Viral Suppression* [25, 34], while another found groups spending less than one month away from their community to have significantly greater odds of *Viral Suppression* compared to mobile groups [27]. No clear association was observed between *Viral Suppression* and occupation, income, substance use or gender‐based violence.

### 3.6 Population‐wide viral suppression

Twenty‐three studies reported *Population‐wide Viral Suppression* and only three performed a statistical comparison of *Population‐wide Viral Suppression* across a demographic or risk strata of interest. A study in Kenya and Uganda found that groups aged 15 to 24 and 25 to 34 had statistically lower *Population‐wide Viral Suppression* compared to groups aged greater than 44 (RD −9.2%; 95% CI −3.6 to −14.9) [36]. The authors found that groups spending one month or longer away from their community had lower risks of *Population‐wide Viral Suppression* compared to groups spending less than one month away from their community, but this finding was not statistically significant (RD 2·6%; 95% CI −0.6 to 5.9%). They also found that groups who had never married had lower probability of *Population‐wide Viral Suppression* compared to groups who were currently married. In Botswana men were less likely to have prevalent *Population‐wide Viral Suppression* compared to women (PR 0.92; 95% CI 0.87 to 0.98). Sixteen to nineteen‐year‐old persons had drastically lower prevalence of *Population‐wide Viral Suppression* compared to those aged 60 or older (PR 0.41; 95% CI 0.29 to 0.59). All groups of mobility (<1 week, 1 to 2 weeks, 3 to 4 weeks, 5 to 12 weeks, and >12 weeks) had lower prevalences of *Population‐wide Viral Suppression* compared to groups spending no time away from their community, with the most mobile group having the lowest prevalence of *Population‐wide Viral Suppression* (PR 0.78; 95% CI 0.64 to 0.95). Groups with higher than senior secondary education had lower prevalence of *Population‐wide Viral Suppression* compared to those with non‐formal education in this study setting (PR 0.81; 95% CI 0.73 to 0.89). Women in a South African study had 2.11‐fold greater odds of *Population‐wide Viral Suppression* relative to men (95% CI 1.69 to 2.63) [27]. They also identified a monotonic decrease in odds of *Population‐wide Viral Suppression* across decreasing 5‐year age bands comparing 45 to 49 to 40 to 44, 35 to 39, 30 to 34, 25 to 29, 20 to 24 and 15 to 19 (OR of 0.94, 0.76, 0.51, 0.32, 0.23 and 0.22 respectively). Married persons had 1.5 greater odds of *Population‐wide Viral Suppression* relative to single persons (95% CI 1.08 to 2.09). However, the study failed to detect an association between *Population‐wide Viral Suppression* and any levels of education or mobility.

### 3.7 Distribution of outcome by age and gender

**Figure 4:**
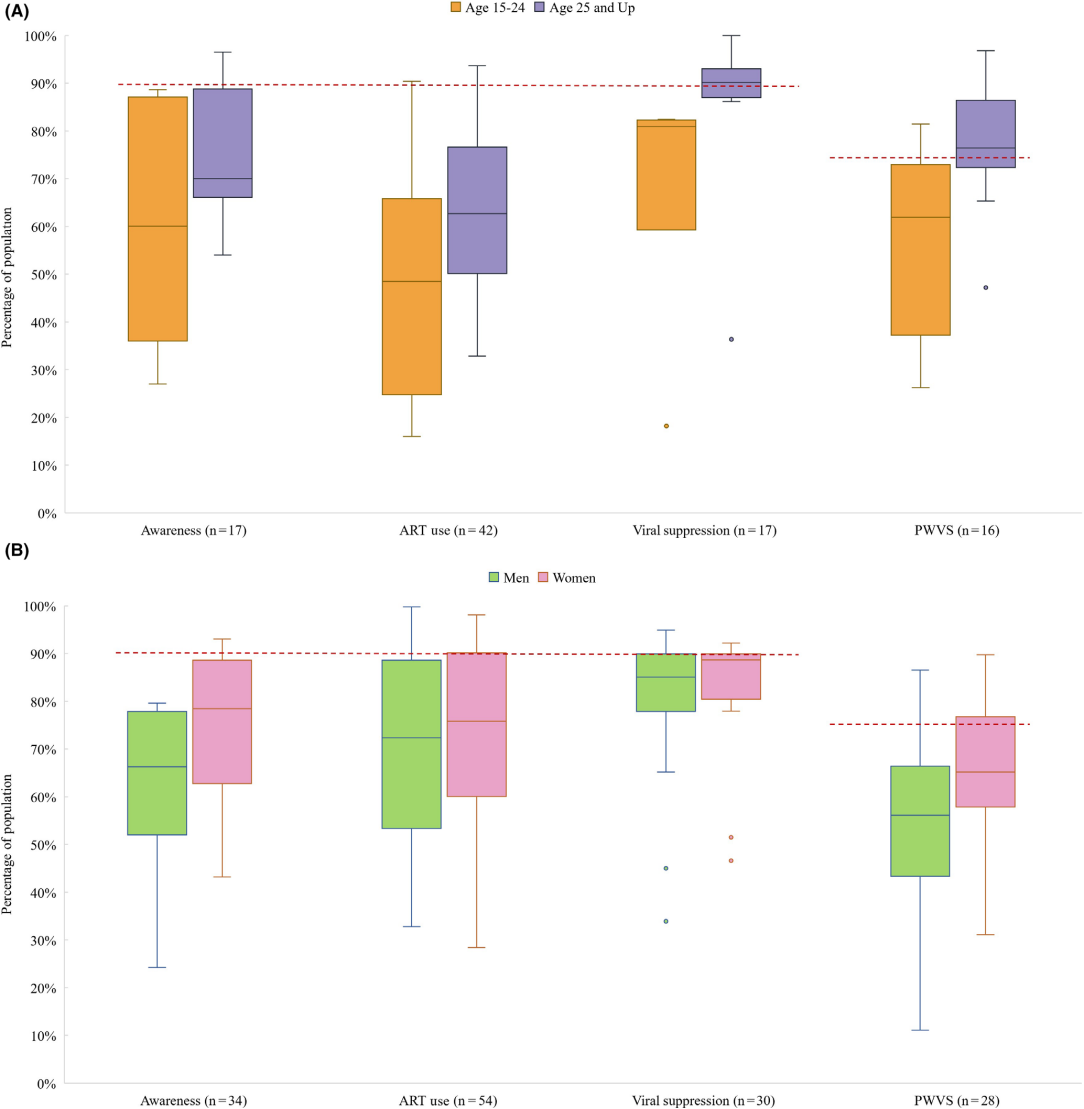
Percentage achieving Awareness of HIV‐positive Status, ART Use, Viral Suppression and Population‐wide Viral Suppression (PWVS) by age group **(A)** and by gender **(B)**. Data in box plots reflect individual studies and are a distribution of study estimates. Results are not weighted by sample size or sampling design, and therefore medians and interquartile ranges and do not represent unbiased estimates of the outcomes for the underlying target populations. Red dashed line represents UNAIDS’ 90% goal for Awareness of HIV‐Positive Status, ART Use, and Viral Suppression, and 73% goal for Population‐wide Viral Suppression. Note that the first three sets of bars in each figure are conditioned on the previous bar, while PWVS is an aggregate estimate. For age, 57% of studies were cross‐sectional, 14% cohort, 21% RCT and 7% case‐control. By gender, 72% are cross‐sectional, 16% are cohort, 9% RCT and 3% are case‐control.

Many included studies stratified count data by age and gender. For age, 17, 42, 17 and 16 studies reported count data by suitable age groups for *Awareness*, *ART Use, Viral Suppression, and PWVS.* Among studies which presented count data, those aged 15 to 25 had lower achievement across the treatment cascade relative to groups aged 25 and up (Figure 4A). The younger group had a lower median of *Awareness, ART Use, Viral Suppression* and *Population‐wide Viral Suppression* of (60‐48‐81‐62) while the older age group had a median achievement of (70‐63‐91‐76). The low outliers for *Viral Suppression* for both age groups are sourced from a study focused on an incarcerated population.

For gender, 34, 54, 30 and 28 studies reported count data for *Awareness*, *ART Use, Viral Suppression and PWVS.* The median of the achievement of each outcome was lesser for men than for women (Figure 4B). Men had median achievement of *Awareness of HIV‐Positive Status*, *ART Use, Viral Suppression* and *Population‐wide Viral Suppression* at (66‐72‐85‐56) versus (78‐76‐89‐65) for women. All low outliers for *Viral Suppression* by gender were sourced from studies set in South Africa.

**Figure 5:**
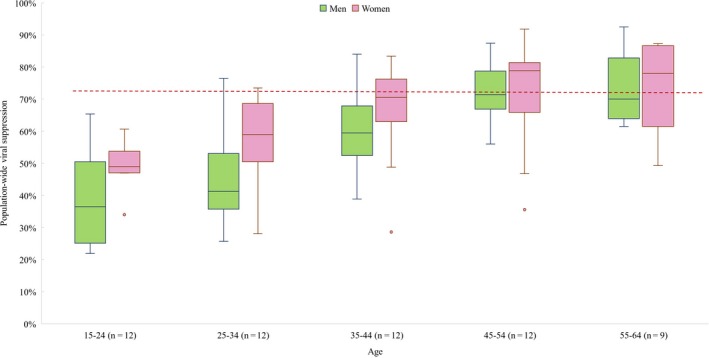
Percentage of population achieving viral suppression by age group and gender. Only PHIA and South African National HIV Prevalence, Incidence, Behaviour, and Communication Survey are included, as they had similar population‐based sampling designs and presented data stratified by similar age bands and gender. Data in box plots reflect individual studies and are a distribution of study estimates. Results are not weighted by sample size or sampling design, and therefore medians and interquartile ranges and do not represent unbiased estimates of the outcomes for the underlying target populations. Red dashed line represents UNAIDS’ 90% goal for Awareness of HIV‐positive Status, ART Use and Viral Suppression, and 73% goal for Population‐wide Viral Suppression.

Eleven PHIA set in Malawi, Zimbabwe, Zambia, Tanzania, Lesotho, eSwatini, Uganda, Cameroon, Cote d'Ivoire, Namibia and Ethiopia, as well as one national survey in South Africa have similar study designs and analyses allowing for compatible groupings of age and gender for *Population‐wide Viral Suppression*. These studies are featured elsewhere in this review, but also allowed for this sub‐analysis. Among these studies, we find women to have higher levels of *Population‐wide Viral Suppression* at each age group compared to men (Figure 5). Furthermore, there is a trend of older age groups having higher achievement of *Population‐wide Viral Suppression* compared to younger age groups within each gender. The median achievement of *Population‐wide Viral Suppression* for women aged 35 and up as well as men aged 45 to 54 have surpassed the 73% UNAIDS goal. All other age‐gender groups have yet to meet this goal, with men aged 15 to 24 having a median achievement of only 37%.

## 4 Discussion

In this systematic review of variables associated with achievement of the HIV treatment cascade, we found heterogeneity in the 90‐90‐90 outcomes across age and gender. Youths and adolescents aged 15 to 24 had a median *Population‐wide Viral suppression* of 62%, while those aged 25 and up had a median achievement of 76% – exceeding the 90‐90‐90 goal. Men had particularly low *Population‐wide Viral suppression* with a median of 56% while women's median achievement was 65%. When disaggregated by age and gender, men aged 15 to 24 had the lowest levels of *Population‐wide Viral suppression* at only 37%, which is roughly half of the UNAIDS target. We also found that mobile populations were significantly less likely to be virally suppressed, a 0.78 prevalence ratio in Botswana, and 0.55‐fold lower odds of viral suppression in South Africa. Patterns across other demographic features such as relationship status, education or occupation were less consistent across included studies, but there were associations between these features and progression through the HIV treatment cascade.

We found no data on measures of risk behaviour across the outcomes of interest, a key gap, as risk behaviours such as condom usage and number of sexual partners are most strongly associated with increased risk of HIV transmission. Ascertainment of these risk behaviours underpins identification of high‐risk groups, which are ideal targets for programmatic intervention. With better information on the heterogeneity of sociodemographics and risk behaviour across the treatment cascade, HIV programme implementers would be better armed to target populations at each cascade element. This could lead to more equitable programmes and more significant decreases in HIV transmission rates.

Although we lack information on directly reported risk behaviour and heterogeneity, there was consistent evidence on some key demographic features. Age and gender have been among the most studied, important and complicated demographic features surrounding HIV risk of transmission in Africa. As of 2018, adolescents and young adults aged 15 to 24, particularly girls in SSA, have been among the highest risk of HIV infection in the world and are estimated to contribute to nearly 20% of all new infections annually [37]. Immigration and mobility patterns have been an emerging area of research for understanding risk of transmission as well as access to testing and treatment services [38, 39].

Our study has important limitations worth mentioning. Like all systematic reviews, our review is subject to publication bias. However, we have sought to primarily report non‐primary objective associations and presume lesser publication pressure and resulting bias in our findings. We also note that we make no restrictions to included study design nor measure of association for inclusion and conducted an extensive grey literature review. While care must be taken in interpreting results, we feel it is important that we highlight the existence of statistical patterns of disparity across the treatment cascade rather than the precise measure of differential achievement.

These findings have critical implications for HIV programmes, donors and implementers. Currently, many national strategic plans focus on aggregate targeting and achievement of the 90‐90‐90 goals with the expectation that their achievement will be accompanied by significant declines in HIV incidence. However, the feasibility of these national plans may be limited, and the likelihood of their achievement may be improved by ensuring HIV programme strategies address underlying sociodemographic heterogeneity in the treatment cascade [40]. Furthermore, given heterogeneity in achieving the 90‐90‐90 goals, there is a need to improve allocative efficiency of available resources. Reaching those groups who are lagging behind in the treatment cascade could increase impact on reducing HIV transmission.

## 5 Conclusions

In conclusion, there was consistent and significant heterogeneity in *Awareness of HIV‐Positive Status*, *ART Use* and *Viral Suppression* by age and sex among adults in sub‐Saharan Africa. For twelve national, population‐based surveys, women had greater achievement along the treatment cascade than men within each age group, and an increasing trend in achievement within each gender across ages. There was limited data on sexual risk behaviour throughout the treatment cascade. This information could not only be useful in the design and targeting of national HIV programmes but could also inform the development of policies on the road to ending AIDS by 2030.

## Competing interests

We declare no competing interests.

## Authors’ contributions

AA and AB conceptualized the study, and all authors contributed to the study protocol. DG, DMT and BK conducted the literature search and screened titles, abstracts and full text articles for inclusion. DG and DMT extracted data from included studies and analysed results. DG generated tables and figures and wrote the first draft of the manuscript. AA, AB, MM, GG, AD and PD provided oversight, technical support, critical feedback and interpretation of results. All authors contributed to writing the manuscript and approved the final version.

## Supporting information


**File S1.** Search terms and strategy.

**File S2.** Studies of demographic heterogeneity of 90‐90‐90 which met inclusion criteria.

**File S3.** Risk of bias assessment results by study and in aggregate.
