# Supplementary material for: Evidence of sociodemographic heterogeneity across the HIV treatment cascade and progress towards 90‐90‐90 in sub‐Saharan Africa – a systematic review and meta‐analysis
Source: J Int AIDS Soc. 2020 Mar 9;23(3):e25470. doi: 10.1002/jia2.25470 (PMC7062634; doi:10.1002/jia2.25470)
Supplement: Supplementary file 1 — File S1. Search terms and strategy. [file JIA2-23-e25470-s001.pdf]

Additional File 1: Search Terms

**MEDLINE**	
1st 90 - *Awareness of HIV-Positive status*	("hiv infections/diagnosis"[Mesh Terms] OR "hiv infections/prevention and control"[Mesh Terms] OR "hiv infections/prevention and control"[tiab] OR "hiv infections/diagnosis"[tiab])<br><br>AND ("diagnosis"[MeSH Major Topic] OR "hiv testing"[tiab] OR serostatus[tiab] OR "hiv status"[tiab] OR "point-of-care test"[tiab] OR "linkage to care"[tiab] OR "rapid test"[tiab])<br><br>AND ("africa south of the sahara"[MeSH Terms] OR subsaharan[tw] OR sub-saharan[tw] OR (sahara[tw] OR sahara'[tw] OR sahara's[tw] OR saharae[tw] OR saharagalago[tw] OR saharai[tw] OR saharal[tw] OR saharam[tw] OR saharan[tw] OR saharan'[tw] OR saharana[tw] OR saharanafrica[tw] OR saharanian[tw] OR saharanpur[tw] OR saharans[tw] OR saharastega[tw] OR saharatm[tw] OR saharatrade[tw] OR saharaui[tw] OR saharawi[tw] OR saharawis[tw]) OR central africa[tw] OR east africa[tw] OR eastern africa[tw] OR south africa[tw] OR southern africa[tw] OR west africa[tw] OR western africa[tw] OR angola[tw] OR Benin[tw] OR Botswana[tw] OR Burkina Faso[tw] OR Burundi[tw] OR cabo verde[tw] OR cameroon[tw] OR central african republic[tw] OR Chad[tw] OR congo[tw] OR Cote d'Ivoire[tw] OR djibouti[tw] OR Eritrea[tw] OR Ethiopia[tw] OR Equatorial Guinea[tw] OR Gabon[tw] OR Gambia[tw] OR Ghana[tw] OR Guinea[tw] OR Guinea-Bissau[tw] OR Ivory Coast[tw] OR Kenya[tw] OR Lesotho[tw] OR liberia[tw] OR mali[tw] OR Malawi[tw] OR Mozambique[tw] OR Mali[tw] OR Mauritania[tw] OR Namibia[tw] OR Niger[tw] OR Nigeria[tw] OR madagascar[tw] OR mauritius[tw] OR Rwanda[tw] OR Somalia[tw] OR Senegal[tw] OR Sierra Leone[tw] OR Sudan[tw] OR South Sudan[tw] OR "swaziland"[tw] OR tanzania[tw] OR togo[tw] OR uganda[tw] OR zambia[tw] OR zimbabwe[tw])<br><br>AND 2014[PDAT] : 2018[PDAT]<br><br>NOT (Letter[pt] OR Editorial[pt] OR Review[pt] OR News[pt] OR Meta-Analysis[pt] OR Guideline[pt])
2nd 90 - *ART Use* among those aware of status	("hiv infections/diagnosis"[Mesh Terms] OR "hiv infections/prevention and control"[Mesh Terms] OR "hiv infections/prevention and control"[tiab] OR "hiv infections/diagnosis"[tiab])<br><br>AND (HAART[tiab] OR ART [tiab] OR ARV [tiab] OR cART [tiab] OR antiretroviral [tiab] OR "HIV treatment"[tiab] OR "linkage to care"[tiab] OR "treatment as prevention"[tiab] OR "access to care"[tiab] OR "treatment access"[tiab])<br><br>AND ("africa south of the sahara"[MeSH Terms] OR subsaharan[tw] OR sub-saharan[tw] OR (sahara[tw] OR sahara'[tw] OR sahara's[tw] OR saharae[tw] OR saharagalago[tw] OR saharai[tw] OR saharal[tw] OR saharam[tw] OR saharan[tw] OR saharan'[tw] OR saharana[tw] OR saharanafrica[tw] OR saharanian[tw] OR saharanpur[tw] OR saharans[tw] OR saharastega[tw] OR saharatm[tw] OR saharatrade[tw] OR saharaui[tw] OR saharawi[tw] OR saharawis[tw]) OR central africa[tw] OR east africa[tw] OR eastern africa[tw] OR south africa[tw] OR southern africa[tw] OR west africa[tw] OR western africa[tw] OR angola[tw] OR Benin[tw] OR Botswana[tw] OR Burkina Faso[tw] OR Burundi[tw] OR cabo verde[tw] OR

	cameroon[tw] OR central african republic[tw] OR Chad[tw] OR congo[tw] OR Cote d'Ivoire[tw] OR djibouti[tw] OR Eritrea[tw] OR Ethiopia[tw] OR Equatorial Guinea[tw] OR Gabon[tw] OR Gambia[tw] OR Ghana[tw] OR Guinea[tw] OR Guinea-Bissau[tw] OR Ivory Coast[tw] OR Kenya[tw] OR Lesotho[tw] OR liberia[tw] OR mali[tw] OR Malawi[tw] OR Mozambique[tw] OR Mali[tw] OR Mauritania[tw] OR Namibia[tw] OR Niger[tw] OR Nigeria[tw] OR madagascar[tw] OR mauritius[tw] OR Rwanda[tw] OR Somalia[tw] OR Senegal[tw] OR Sierra Leone[tw] OR Sudan[tw] OR South Sudan[tw] OR "swaziland"[tw] OR tanzania[tw] OR togo[tw] OR uganda[tw] OR zambia[tw] OR zimbabwe[tw])<br><br>AND 2014[PDAT] : 2018[PDAT]<br><br>NOT (Letter[pt] OR Editorial[pt] OR Review[pt] OR News[pt] OR Meta-Analysis[pt] OR Guideline[pt]
3rd 90 - *Viral Suppression* among those on ART	("hiv infections/diagnosis"[Mesh Terms] OR "hiv infections/prevention and control"[Mesh Terms] OR "hiv infections/prevention and control"[tiab] OR "hiv infections/diagnosis"[tiab])<br><br>AND ("viral suppression"[tiab] OR undetected[tiab] OR "viral load"[tiab] OR "virologic failure"[tiab] OR unsuppressed[tiab] OR "treatment failure"[tiab] OR "treatment adherence"[tiab] OR "ART adherence"[tiab])<br><br>AND ("africa south of the sahara"[MeSH Terms] OR subsaharan[tw] OR sub-saharan[tw] OR (sahara[tw] OR sahara'[tw] OR sahara's[tw] OR saharae[tw] OR saharagalago[tw] OR saharai[tw] OR saharal[tw] OR saharam[tw] OR saharan[tw] OR saharan'[tw] OR saharana[tw] OR saharanafrica[tw] OR saharanian[tw] OR saharanpur[tw] OR saharans[tw] OR saharastega[tw] OR saharatm[tw] OR saharatrade[tw] OR saharaui[tw] OR saharawi[tw] OR saharawis[tw]) OR central africa[tw] OR east africa[tw] OR eastern africa[tw] OR south africa[tw] OR southern africa[tw] OR west africa[tw] OR western africa[tw] OR angola[tw] OR Benin[tw] OR Botswana[tw] OR Burkina Faso[tw] OR Burundi[tw] OR cabo verde[tw] OR cameroon[tw] OR central african republic[tw] OR Chad[tw] OR congo[tw] OR Cote d'Ivoire[tw] OR djibouti[tw] OR Eritrea[tw] OR Ethiopia[tw] OR Equatorial Guinea[tw] OR Gabon[tw] OR Gambia[tw] OR Ghana[tw] OR Guinea[tw] OR Guinea-Bissau[tw] OR Ivory Coast[tw] OR Kenya[tw] OR Lesotho[tw] OR liberia[tw] OR mali[tw] OR Malawi[tw] OR Mozambique[tw] OR Mali[tw] OR Mauritania[tw] OR Namibia[tw] OR Niger[tw] OR Nigeria[tw] OR madagascar[tw] OR mauritius[tw] OR Rwanda[tw] OR Somalia[tw] OR Senegal[tw] OR Sierra Leone[tw] OR Sudan[tw] OR South Sudan[tw] OR "swaziland"[tw] OR tanzania[tw] OR togo[tw] OR uganda[tw] OR zambia[tw] OR zimbabwe[tw])<br><br>AND 2014[PDAT] : 2018[PDAT]<br><br>NOT (Letter[pt] OR Editorial[pt] OR Review[pt] OR News[pt] OR Meta-Analysis[pt] OR Guideline[pt])
90-90-90 – *Population-wide viral suppression*	("hiv infections/diagnosis"[Mesh Terms] OR "hiv infections/prevention and control"[Mesh Terms] OR "hiv infections/prevention and control"[tiab] OR "hiv infections/diagnosis"[tiab])<br><br>AND ("90-90-90"[tiab] OR cascade[tiab] OR "fast-track"[tiab] OR "test and start"[tiab] OR "treatment as prevention"[tiab])

	AND ("africa south of the sahara"[MeSH Terms] OR subsaharan[tw] OR sub-saharan[tw] OR (sahara[tw] OR sahara'[tw] OR sahara's[tw] OR saharae[tw] OR saharagalago[tw] OR saharai[tw] OR saharal[tw] OR saharam[tw] OR saharan[tw] OR saharan'[tw] OR saharana[tw] OR saharanafrica[tw] OR saharanian[tw] OR saharanpur[tw] OR saharans[tw] OR saharastega[tw] OR saharatm[tw] OR saharatrade[tw] OR saharaui[tw] OR saharawi[tw] OR saharawis[tw]) OR central africa[tw] OR east africa[tw] OR eastern africa[tw] OR south africa[tw] OR southern africa[tw] OR west africa[tw] OR western africa[tw] OR angola[tw] OR Benin[tw] OR Botswana[tw] OR Burkina Faso[tw] OR Burundi[tw] OR cabo verde[tw] OR cameroon[tw] OR central african republic[tw] OR Chad[tw] OR congo[tw] OR Cote d'Ivoire[tw] OR djibouti[tw] OR Eritrea[tw] OR Ethiopia[tw] OR Equatorial Guinea[tw] OR Gabon[tw] OR Gambia[tw] OR Ghana[tw] OR Guinea[tw] OR Guinea-Bissau[tw] OR Ivory Coast[tw] OR Kenya[tw] OR Lesotho[tw] OR liberia[tw] OR mali[tw] OR Malawi[tw] OR Mozambique[tw] OR Mali[tw] OR Mauritania[tw] OR Namibia[tw] OR Niger[tw] OR Nigeria[tw] OR madagascar[tw] OR mauritius[tw] OR Rwanda[tw] OR Somalia[tw] OR Senegal[tw] OR Sierra Leone[tw] OR Sudan[tw] OR South Sudan[tw] OR "swaziland"[tw] OR tanzania[tw] OR togo[tw] OR uganda[tw] OR zambia[tw] OR zimbabwe[tw])<br><br>AND 2014[PDAT] : 2018[PDAT]<br><br>NOT (Letter[pt] OR Editorial[pt] OR Review[pt] OR News[pt] OR Meta-Analysis[pt] OR Guideline[pt])
**Embase**	
1st 90 - *Awareness of HIV-Positive status*	('human immunodeficiency virus infection'/exp OR 'human immunodeficiency virus infection')<br><br>AND ('diagnostic test'/mj OR serostatus OR 'point-of-care test' OR 'linkage to care'/mj OR 'rapid test'/mj)<br><br>AND ('africa'/exp OR 'africa')<br><br>NOT 'letter' NOT 'editorial' NOT 'review' NOT 'meta analysis'<br><br>AND [embase]/lim NOT ([embase]/lim AND [medline]/lim)<br><br>AND (2014:py OR 2015:py OR 2016:py OR 2017:py OR 2018:py)
2nd 90 - *ART Use* among those aware of status	('human immunodeficiency virus infection'/mj)<br><br>AND (haart OR art OR cart OR antiretroviral OR 'hiv treatment' OR 'linkage to care' OR 'treatment as prevention' OR 'access to care' OR 'treatment access')<br><br>AND ('africa'/exp OR 'africa')<br><br>NOT 'letter' NOT 'editorial' NOT 'review' NOT 'meta analysis'<br><br>AND [embase]/lim NOT ([embase]/lim AND [medline]/lim)<br><br>AND (2014:py OR 2015:py OR 2016:py OR 2017:py OR 2018:py)

3rd 90 - *Viral Suppression* among those on ART	('human immunodeficiency virus infection'/mj)<br><br>AND ('viral suppression' OR undetected OR 'viral load' OR 'virologic failure' OR unsuppressed OR 'treatment failure' OR 'treatment adherence' OR 'art adherence')<br><br>AND ('africa'/exp OR 'africa')<br><br>NOT 'letter' NOT 'editorial' NOT 'review' NOT 'meta analysis'<br><br>AND [embase]/lim NOT ([embase]/lim AND [medline]/lim)<br><br>AND (2014:py OR 2015:py OR 2016:py OR 2017:py OR 2018:py)
90-90-90 - *Population-wide viral suppression*	('human immunodeficiency virus infection'/mj)<br><br>AND ('90-90-90' OR cascade OR 'fast-track' OR 'test and start' OR 'treatment as prevention')<br><br>AND ('africa'/exp OR 'africa')<br><br>NOT 'letter' NOT 'editorial' NOT 'review' NOT 'meta analysis'<br><br>AND [embase]/lim NOT ([embase]/lim AND [medline]/lim)<br><br>AND (2014:py OR 2015:py OR 2016:py OR 2017:py OR 2018:py)
