# Supplementary material for: Evidence of sociodemographic heterogeneity across the HIV treatment cascade and progress towards 90‐90‐90 in sub‐Saharan Africa – a systematic review and meta‐analysis
Source: J Int AIDS Soc. 2020 Mar 9;23(3):e25470. doi: 10.1002/jia2.25470 (PMC7062634; doi:10.1002/jia2.25470)
Supplement: Supplementary file 2 — File S2. Studies of demographic heterogeneity of 90‐90‐90 which met inclusion criteria. [file JIA2-23-e25470-s002.pdf]

Additional File 2: Studies of demographic heterogeneity of 90-90-90 which met inclusion criteria

						Outcome(s) Reported				Outcome(s) Stratified by:						
**First author (publication year)**	**Country(s)**	**Study period**	**Study design**	**Sampling design**	**Sample size**	**Awareness**	**ART Use**	**Viral Suppression**	**PWVS**	**Age**	**Gender**	**Education**	**Income or wealth**	**Relationship status**	**Mobility**	**Occupation**
**Peer-reviewed literature**																
ME. Charurat (2015)[1]	Nigeria	Mar. 2013 - Aug. 2014	C	RDS	186		X	X		X		X		X		X
L. Myer (2015)[2]	South Africa	Apr. 2013 - Jun. 2014	CS	CFB	1521		X	X		X		X		X		X
NE. Rosenberg (2015)[3]	Malawi	Mar. 2014 - Oct. 2014	RCT	CFB	200	X										
JM. Baeten (2016)[4]	Kenya, Uganda	Nov. 2012 - Aug. 2014	C	CB	1013				X							
RV. Barnabas (2016)[5]	South Africa, Uganda	Jun. 2013 - Feb. 2015	RCT	CB	15322	X	X	X								
S. Boyer (2016)[6]	South Africa	2012 - 2015	RCT	PB	514		X			X	X	X	X	X		X
MM. Chanda (2017)[7]	Zambia	Sept. 2016 - Oct. 2016	RCT	CB	965		X									
SE. Collins (2016)[8]	Rwanda	Apr. 2014 - Sept. 2014	RCT	CFB	150			X		X	X					
T. Gaolathe (2016)[9]	Botswana	Oct. 2013 - Nov. 2015	RCT	PB	3596	X	X	X	X	X	X	X	X	X	X	X
A. Grobler (2017)[10]	South Africa	Jun. 2014 - Jun. 2015	CS	PB	9812	X	X	X	X	X	X	X		X	X	
KE. Lancaster (2016)[11]	Malawi	Jul. 2014 - Sep. 2014	CS	VB	138	X										
J. Manne-Goehler (2016)[12]	South Africa	Nov. 2014 - Nov. 2015	C	CB	4767		X									
T. Nakanyala (2016)[13]	Namibia	Dec. 2014 - Jul. 2015	CS	CB	2163	X	X	X	X	X	X					
A. Rao (2016)[14]	South Africa	Oct. 2014 - Apr. 2015	CS	RDS	391	X				X		X	X	X		
S. Stahlman (2016)[15]	Lesotho	Feb. 2014 - Jun. 2014	CS	RDS	318	X										
S. Ahmed (2017)[16]	Malawi	Jul. 2014 - Apr. 2015	C	CB	711	X	X			X						
S. Asiimwe (2017)[17]	Uganda	Oct. 2015 - Mar. 2016	CS	CB	43696		X									
B. Elul (2017)[18]	Mozambique	Apr. 2013 - Jun. 2016	RCT	CFB	2004		X									
AG. Flynn (2017)[19]	Uganda	2006 - 2016	C	CFB	1353	X	X									
MK. Grabowski (2017)[20]	Uganda	Apr. 1999 - Sep. 2016	C	PB	33937		X		X		X					
R. Hayes (2017)[21]	Zambia	Dec. 2013 - Jun. 2015	RCT	PB	101102	X	X			X	X					
CJ. Hoffmann (2017)[22]	South Africa	Mar. 2013 - Oct. 2014	RCT	CFB	2558		X									
D. Kerrigan (2017)[23]	Tanzania	Oct. 2015-Apr. 2016	CS	VB	496	X	X	X	X	X		X		X	X	X
A. Laher (2017)[24]	South Africa	Mar. 2016-Aug. 2016	C	CFB	159			X								

First author (publication year)	Country(s)	Study period	Study design	Sampling design	Sample size	Outcome(s) Reported				Outcome(s) Stratified by:						
						Awareness	ART Use	Viral Suppression	PWVS	Age	Gender	Education	Income or wealth	Relationship status	Mobility	Occupation
CE. Lyons (2017)[25]	Senegal	Sep. 2013 - Sep. 2016	C	RDS	1482	X	X	X								
R. Mafigiri (2017)[26]	Uganda	2013 - 2014	CS	CB	792		X				X					
B. Myburgh (2017)[27]	South Africa	Oct. 2013 - Jun. 2014	CS	CFB	100		X									
K. Ortblad (2017)[28]	Uganda	Oct. 2016 - 2017	RCT	CB	960		X									
M. Petersen (2017)[29]	Kenya, Uganda	Jun. 2013 - Jun. 2016	RCT	PB	77774	X	X	X	X	X	X	X	X	X	X	X
S. Phiri (2017)[30]	Malawi	Nov. 2013 - Nov. 2014	RCT	CFB	1269		X	X								
NE. Rosenberg (2017)[31]	Malawi	Dec. 2015 - Aug. 2016	C	CFB	137	X										
EC. Ruria (2017)[32]	Kenya	Jul. 2015 - Dec. 2016	C	CFB	952		X									
E. Ruzagira (2017)[33]	Uganda	Mar. 2015 - Mar. 2016	RCT	CB	412	X	X									
S. Schwartz (2017)[34]	South Africa	Oct. 2014 - Apr. 2015	CS	RDS	410	X	X	X								
A. Vos (2017)[35]	South Africa	Apr. - May 2016	CS	CB	201		X									
L. Vu (2017)[36]	Uganda	Oct. 2014 - Sep. 2015	C	CB	473		X									
K. Brown (2018)[37]	Zimbabwe, Malawi, Zambia, Uganda, Swaziland, Tanzania, Lesotho	2015 - 2017	CS	PB	28152	X	X	X	X							
MB. Chagomerana (2018)[38]	Malawi	Jun. 2015 - Nov. 2016	C	CFB	299			X				X		X		
RKD. Ephraim (2018)[39]	Ghana	Jan. 2014 - Apr. 2014	CC	CFB	110		X			X	X					
B. Hansoti (2018)[40]	South Africa	Sep. 2016 - Nov. 2016	CS	CFB	1714	X					X					
B. Hansoti (2018)[41]	South Africa	Sep. 2016 - Nov. 2016	CS	CFB	2100			X			X					
CB. Holmes (2018)[42]	Zambia	Aug. 2013 - Jul. 2015	CS	CFB	165464		X				X					
F. Moyo (2018)[43]	South Africa	May 2016 - Sep. 2016	CS	CFB	367		X	X		X						
A. Mujugira (2018)[44]	Kenya, Uganda	Nov. 2012 - Jun. 2016	RCT	CFB	904			X			X					
CM. North (2018)[45]	Uganda	Dec. 2013 - Dec. 2018	C	CFB	269			X								
S. Okawa (2018)[46]	Zambia	Apr. 2014 - Jul. 2014	CS	CFB	175		X				X					
J. Ousley (2018)[47]	Kenya, Democratic Republic of Congo	Jan. - Mar. 2015 (Kenya), May - Jul. 2017 (DRC)	CS	CFB	707		X	X		X	X					
M. Seyoum (2018)[48]	Ethiopia	Feb. 2017 - May 2017	CS	CFB	200		X			X	X	X		X		
JO. Ugboaja (2018)[49]	Nigeria	Jan. 2016 - Jan. 2017	CS	CFB	110		X									
Y. Zhang (2018)[50]	Kenya, Malawi, South Africa	Jul. 2015 - Apr. 2018	RCT	CFB	183		X			X	X				X	

						Outcome(s) Reported				Outcome(s) Stratified by:						
**First author (publication year)**	**Country(s)**	**Study period**	**Study design**	**Sampling design**	**Sample size**	**Awareness**	**ART Use**	**Viral Suppression**	**PWVS**	**Age**	**Gender**	**Education**	**Income or wealth**	**Relationship status**	**Mobility**	**Occupation**
**Grey literature**																
RA. Ayanga (2015)[51]	Uganda	Nov. 2014 - Jan. 2015	C	CFB	1008			X								
V. Jain (2015)[52]	Uganda	2014	CS	CB	4897				X							
J. Rhein (2015)[53]	Uganda	Aug. 2013 - Aug. 2014	C	CFB	165		X									
SR. Schwartz (2015)[54]	South Africa	Jul. 2013 - Sep. 2014	C	CFB	126		X	X								
SJ. Aston (2016)[55]	Malawi	May 2013 - Jan. 2015	C	CFB	459	X	X									
I. Casavant (2016)[56]	Mozambique	2014 - 2015	CS	PB	25344	X										
A Kharsany (2016)[57]	South Africa	2014 - 2015	CS	CB	9812				X	X	X					
M. Lahuerta (2016)[58]	Mali	Oct. 2014 - Feb. 2015	CS	RDS	552	X	X									
WB. MacLeod (2016)[59]	South Africa	Apr. 2014 - Mar. 2015	CS	CFB	2199890			X								
PHIA (2016)[60]	Malawi	Nov. 2015 - Aug. 2016	CS	PB	NA	X	X	X	X	X	X					
PHIA (2016)[61]	Tanzania	Oct. 2016 - Aug. 2017	CS	PB	NA	X	X	X	X	X	X					
PHIA (2016)[62]	Uganda	Aug. 2016 - Mar. 2017	CS	PB	NA	X	X	X	X	X	X					
PHIA (2016)[63]	Zambia	Mar. 2016 - Aug. 2016	CS	PB	NA	X	X	X	X	X	X					
PHIA (2016)[64]	Zimbabwe	Oct. 2015 - Aug. 2016	CS	PB	NA	X	X	X	X							
M. Ribakare (2016)[65]	Rwanda	Jan. 2014 - Dec. 2014	CS	CFB	117226			X								
B. Tippett Barr (2016)[66]	Malawi	Sep. 2013 – Sep. 2016	C	CFB	1851	X	X									
L. Abuogi (2017)[67]	Kenya	Sep. 2014 - Feb. 2016	C	CFB	164		X	X								
G. Breton (2017)[68]	Burundi, Cameroon, Guinea, Cote d'Ivoire	Aug. 2014 - Mar. 2016	C	CFB	31286			X								
C. Couderc (2017)[69]	Mali, Cote d'Ivoire, Togo, Burkina Faso	Jun. 2015 - Jan. 2017	C	CB	679		X									
AB. Kharsany (2017)[70]	South Africa	Jun. 2014 - Jun. 2016	CS	PB	9812		X	X			X					
SM. Kiene (2017)[71]	Uganda	Dec. 2015 – Dec. 2016	CS	PB	5401	X	X	X	X							
T. Malaba (2017)[72]	South Africa	Apr. 2015 - Oct. 2016	C	CFB	1060		X									
SC. Mendelsohn (2017)[73]	Malawi	Jun. 2014 - Dec. 2014	C	VB	4166		X	X								
N. Mutenda (2017)[74]	Namibia	Jan. 2014 - Mar. 2015	C	CFB	8366			X								
H. Ndagire (2017)[75]	Uganda	Oct. 2014 - Jun. 2016	C	VB	2815		X	X								

						Outcome(s) Reported				Outcome(s) Stratified by:						
**First author (publication year)**	**Country(s)**	**Study period**	**Study design**	**Sampling design**	**Sample size**	**Awareness**	**ART Use**	**Viral Suppression**	**PWVS**	**Age**	**Gender**	**Education**	**Income or wealth**	**Relationship status**	**Mobility**	**Occupation**
S. Nicholas (2017)[76]	Uganda	Sept. 2013 - Nov. 2016	C	CFB	9305			X								
A. Nikuze (2017)[77]	Kenya	Feb. 2016 - Jul. 2016	CS	CFB	3585	X	X	X								
M. Nsumba (2017)[78]	Uganda	Jan. 2015 - Dec. 2016	C	CFB	5867			X		X	X					
P. Olang'o (2017)[79]	Kenya	Nov. 2015 - Sep. 2016	C	CFB	5290		X	X								
I. Pathmanathan (2017)[80]	Swaziland	Jul. 2014 - Sept. 2014	C	CFB	466		X									
PHIA (2017)[81]	Lesotho	Nov. 2016 - May 2017	CS	PB	14028	X	X	X	X	X	X					
PHIA (2017)[82]	Eswatini	Aug. 2016 - Mar. 2017	CS	PB	10879	X	X	X	X	X	X					
S. Dadabhai (2018)[83]	Malawi	Jan. 2016 - Sep. 2017	C	CFB	1278			X								
C. Hoffmann (2018)[84]	South Africa	Apr. 2016 - Jun. 2016	C	CFB	121	X	X									
HSRC (2018)[85]	South Africa	Jan. 2017 - Dec. 2017	CS	PB		X	X	X	X	X	X					
LT. Matthews (2018)[86]	South Africa, Uganda	2015 - 2017	C	CFB	439				X							
PHIA (2018)[87]	Cameroon	Jul. 2017 - Feb. 2018	CS	PB		X	X	X	X	X	X					
PHIA (2018)[88]	Cote d'Ivoire	Aug. 2017 - Mar. 2018	CS	PB		X	X	X	X	X	X					
PHIA (2018)[89]	Namibia	Jun. - Dec. 2017	CS	PB		X	X	X	X	X	X					
PHIA (2018)[90]	Ethiopia	Oct. 2017 - Apr. 2018	CS	PB		X	X	X	X	X	X					
JT. Price (2018)[91]	Zambia	Aug. 2015 - Aug. 2017	C	CFB	1425		X	X	X							
E. Zielinski-Gutirrez (2018)[92]	Kenya	Aug. 2014 - Mar. 2015	CS	CB	3462		X									

PHIA: Population-based HIV impact assessment
HSRC: Human sciences research council
Study design: C: Cohort, CS: Cross-sectional, RCT: randomized controlled trial, CC: case-control.
Sample design: RDS: respondent-driven sample, CFB: clinic- or facility-based, CB: community-based, PB: population-based, VB: venue-based
PWVS: Population-wide viral suppression

References

1. Charurat M, Emmanuel B, Akolo C, Keshinro B, Nowak R, Kennedy S, et al. Uptake of treatment as prevention for HIV and continuum of care among HIV-positive men who have sex with men in Nigeria. *J Acquir Immune Defic Syndr*. 2015;**68**(Suppl 2):S114-23.

2. Myer L, Phillips T, Hsiao N, Zerbe A, Petro G, Bekker L, et al. Plasma viraemia in HIV-positive pregnant women entering antenatal care in South Africa. *J Int AIDS Soc*. 2015;**18**:20045.

3. Rosenberg N, Mtande T, Saidi F, Stanley C, Jere E, Paile L, et al. Recruiting male partners for couple HIV testing and counselling in Malawi's option B+ programme: An unblinded randomised controlled trial. *Lancet HIV*. 2015;**2**(11):e483-91.

4. Baeten J, Heffron R, Kidoguchi L, Mugo N, Katabira E, Bukusi E, et al. Integrated delivery of antiretroviral treatment and pre-exposure prophylaxis to HIV-1-serodiscordant couples: A prospective implementation study in Kenya and Uganda. *PLoS Med*. 2016;**13**(8):e1002099.

5. Barnabas R, van Rooyen H, Tumwesigye E, Brantley J, Baeten J, van Heerden A, et al. Uptake of antiretroviral therapy and male circumcision after community-based HIV testing and strategies for linkage to care versus standard clinic referral: a multisite, open-label, randomised controlled trial in South Africa and Uganda. *Lancet HIV*. 2016;**3**(5):e212-20.

6. Boyer S, Iwuji C, Gosset A, Protopopescu C, Okesola N, Plazy M, et al. Factors associated with antiretroviral treatment initiation amongst HIV-positive individuals linked to care within a universal test and treat programme: early findings of the ANRS 12249 TasP trial in rural South Africa. *AIDS Care*. 2016;**28**(Suppl 3):39–51.

7. Chanda M, Ortblad K, Mwale M, Chongo S, Kanchele C, Kamungoma N, et al. HIV self-testing among female sex workers in Zambia: A cluster randomized controlled trial. *PLoS Med*. 2017;**14**(11):e1002442.

8. Collins S, Grant P, Uwinkindi F, Talbot A, Seruyange E, Slamowitz D, et al. A randomized switch from nevirapine-based antiretroviral therapy to single tablet rilpivirine/emtricitabine/tenofovir disoproxil fumarate in virologically suppressed human immunodeficiency virus-1-infected Rwandans. *Open Forum Infect Dis*. 2016;**3**(3):ofw141.

9. Gaolathe T, Wirth K, Holme M, Makhema J, Moyo S, Chakalisa U, et al. Botswana's progress toward achieving the 2020 UNAIDS 90-90-90 antiretroviral therapy and virological suppression goals: A population-based survey. *Lancet HIV*. 2016 May;**3**(5):e221–30.

10. Grobler A, Cawood C, Khanyile D, Puren A, Kharsany A. Progress of UNAIDS 90-90-90 targets in a district in KwaZulu-Natal, South Africa, with high HIV burden, in the HIPSS study: A household-based complex multilevel community survey. *Lancet HIV*. 2017;**4**(11 PG-505–513):e505–13.

11. Lancaster K, Go V, Lungu T, Mmodzi P, Hosseinipour M, Chadwick K, et al. Substance use and HIV infection awareness among HIV-infected female sex workers in Lilongwe, Malawi. *Int J Drug Policy*. 2016;**30**:124–31.

12. Manne-Goehler J, Montana L, Gomez-Olive X, Wade A, Tollman S, Gaziano T, et al. HIV infection, art use, and access to care for NCDs in Agincourt, South Africa. *Top Antivir Med*. 2016;**24**(E-1):281–2.

13. Nakanyala T, Patel S, Sawadogo S, Maher A, Banda K, Wolkon A, et al. How close to 90-90-90? Measuring undiagnosed HIV infection, ART use and viral suppression in a community-based sample from Namibia's highest prevalence region. In: Proceedings of the 2016 International AIDS Conference. Durban, South Africa; 2016.

14. Rao A, Baral S, Phaswana-Mafuya N, Lambert A, Kose Z, Mcingana M, et al. Pregnancy intentions and safer pregnancy knowledge among female sex workers in Port Elizabeth, South Africa. *Obs Gynecol*. 2016;**128**(1):15–21.

15. Stahlman S, Johnston L, Yah C, Ketende S, Maziya S, Trapence G, et al. Respondent-driven sampling as a recruitment method for men who have sex with men in southern sub-Saharan Africa: A cross-sectional analysis by wave. *Sex Transm Infect*. 2016;**92**(4):292–8.

16. Ahmed S, Sabelli R, Simon K, Rosenberg N, Kavuta E, Harawa M, et al. Index case finding facilitates identification and linkage to care of children and young persons living with HIV/AIDS in Malawi. *Trop Med Int Heal*. 2017;**22**(8):1021–9.

17. Asiimwe S, Ross J, Arinaitwe A, Tumusiime O, Turyamureeba B, Roberts D, et al. Expanding HIV testing and linkage to care in southwestern Uganda with community health extension workers. *J Int AIDS Soc*. 2017;**20**(Suppl 4):21633.

18. Elul B, Lamb M, Lahuerta M, Abacassamo F, Ahoua L, Kujawski S, et al. A combination intervention strategy to improve linkage to and retention in HIV care following diagnosis in Mozambique: A cluster-randomized study. *PLoS Med*. 2017;**14**(11):e1002433.

19. Flynn A, Meya D, Hullsiek K, Rhein J, Williams D, Musubire A, et al. Evolving failures in the delivery of human immunodeficiency virus care: lessons from a Ugandan meningitis cohort 2006-2016. *Open Forum Infect Dis*. 2017;**4**(2):ofx077.

20. Grabowski M, Serwadda D, Gray R, Nakigozi G, Kigozi G, Kagaayi J, et al. HIV prevention efforts and incidence of HIV in Uganda. *N Engl J Med*. 2017;**377**(22):2154–66.

21. Hayes R, Floyd S, Schaap A, Shanaube K, Bock P, Sabapathy K, et al. A universal testing and treatment intervention to improve HIV control: One-year results from intervention communities in Zambia in the HPTN 071 (PopART) cluster-randomised trial. *PLoS Med*. 2017;**14**(5 PG):e1002292.

22. Hoffmann C, Mabuto T, Ginindza S, Fielding K, Kubeka G, Dowdy D, et al. Strategies to accelerate HIV Care and antiretroviral therapy initiation after HIV diagnosis: a randomized trial. *J Acquir Immune Defic Syndr*. 2017;**75**(5):540–7.

23. Kerrigan D, Mbwambo J, Likindikoki S, Beckham S, Mwampashi A, Shembilu C, et al. Project Shikamana: Baseline findings from a community empowerment-based combination HIV prevention trial among female sex workers in Iringa, Tanzania. *J Acquir Immune Defic Syndr*. 2017;**74**(Suppl 1):S60–8.

24. Laher A, Ariefdien N, Etlouba Y. HIV prevalence among first-presentation psychotic patients. *HIV Med*. 2017;**19**(4):271–9.

25. Lyons C, Ketende S, Diouf D, Drame F, Liestman B, Coly K, et al. Potential impact of integrated stigma mitigation interventions in improving HIV/AIDS service delivery and uptake for key populations in Senegal. *J Acquir Immune Defic Syndr*. 2017;**74**(Suppl 1):S52–9.

26. Mafigiri R, Matovu J, Makumbi F, Ndyanabo A, Nabukalu D, Sakor M, et al. HIV prevalence and uptake of HIV/AIDS services among youths (15-24 Years) in fishing and neighboring communities of Kasensero, Rakai district, southwestern Uganda. *BMC Public Health*. 2017;**17**(1):251.

27. Myburgh B, Nel R, Lategan-Potgieter R. Implementation of the prevention of mother-to-child transmission (PMTCT) program in the Northern Cape, South Africa. *Curr HIV Res*. 2017;**15**(1):38–45.

28. Ortblad K, Kibuuka Musoke D, Ngabirano T, Nakitende A, Magoola J, Kayiira P, et al. Direct provision versus facility collection of HIV self-tests among female sex workers in Uganda: A cluster-randomized controlled health systems trial. *PLoS Med*. 2017;**14**(11):e1002458.

29. Petersen M, Balzer L, Kwarsiima D, Sang N, Chamie G, Ayieko J, et al. Association of implementation of a universal testing and treatment intervention with HIV diagnosis, receipt of antiretroviral therapy, and viral suppression in east Africa. *JAMA*. 2017;**317**(21):2196–206.

30. Phiri S, Tweya H, van Lettow M, Rosenberg N, Trapence C, Kapito-Tembo A, et al. Impact of facility- and community-based peer support models on maternal uptake and retention in Malawi’s option B+ HIV prevention of mother-to-child transmission program:

A 3-arm cluster randomized controlled trial (PURE Malawi). *J Acquir Immune Defic Syndr*. 2017;**75**(Suppl 2):S140–8.

31. Rosenberg N, Graybill L, Wesevich A, McGrath N, Golin C, Maman S, et al. The impact of couples HIV testing and counseling on consistent condom use among pregnant women and their male partners: An observational study. *J Acquir Immune Defic Syndr*. 2017;**75**(4):417–25.

32. Ruria E, Masaba R, Kose J, Woelk G, Mwangi E, Matu L, et al. Optimizing linkage to care and initiation and retention on treatment of adolescents with newly diagnosed HIV infection. *AIDS*. 2017;**31**(Suppl 3):S253–60.

33. Ruzagira E, Grosskurth H, Kamali A, Baisley K. Brief counselling after home-based HIV counselling and testing strongly increases linkage to care: a cluster-randomized trial in Uganda. *J Int AIDS Soc*. 2017;**20**(2).

34. Schwartz S, Lambert A, Phaswana-Mafuya N, Kose Z, McIngana M, Holland C, et al. Engagement in the HIV care cascade and barriers to antiretroviral therapy uptake among female sex workers in Port Elizabeth, South Africa: Findings from a respondent-driven sampling study. *Sex Transm Infect*. 2017;**93**(4):290–6.

35. Vos A, Varkila M, Tempelman H, Devillé W, Barth R, Grobbee D, et al. The influence of HIV infection on pulmonary function in a rural african population. *Top Antivir Med*. 2017;**25**(1):276s-277s.

36. Vu L, Burnett-Zieman B, Banura C, Okal J, Elang M, Ampwera R, et al. Increasing uptake of HIV, sexually transmitted infection, and family planning services, and reducing HIV-related risk behaviors among youth living with HIV in Uganda. *J Adolesc Heal*. 2017;**60**(Suppl 2):S22–8.

37. Brown K, Williams D, Kinchen S, Saito S, Radin E, Patel H, et al. Status of HIV epidemic control among adolescent girls and young women aged 15-24 years - seven African countries, 2015-2017. *MMWR Morb Mortal Wkly Rep*. 2018;**67**(1):29–32.

38. Chagomerana M, Miller W, Tang J, Hoffman I, Mthiko B, Phulusa J, et al. Optimizing prevention of HIV mother to child transmission: Duration of antiretroviral therapy and viral suppression at delivery among pregnant Malawian women. *PLoS One*. 2018;**13**(4):e0195033.

39. Ephraim R, Ahadzie J, Adu P, Boachie J, Agbodzakey H, Adoba P, et al. Abnormal coagulation profile in people living with HIV-AIDS on combined antiretroviral therapy: findings from a case-control study in the Ho municipality, Ghana. *Pan Afr Med J*. 2018;**29**(121).

40. Hansoti B, Stead D, Parrish A, Reynolds S, Redd A, Whalen M, et al. HIV testing in a

South African emergency department: A missed opportunity. *PLoS One*. 2018;**13**(3):e0193858.

41. Hansoti B, Eisenberg A, Stead D, Mvandaba N, Patel E, Parrish A, et al. High incidence and burden of HIV infection in East London, South Africa. *Top Antivir Med*. 2018;**26**:424s.

42. Holmes C, Sikazwe I, Sikombe K, Eshun-Wilson I, Czaicki N, Beres L, et al. Estimated mortality on HIV treatment among active patients and patients lost to follow-up in 4 provinces of Zambia: Findings from a multistage sampling-based survey. *PLoS Med*. 2018;**15**(1):e1002489.

43. Moyo F, Haeri Mazanderani A, Bhardwaj S, Mhlongo O, Kufa T, Ng'Oma K, et al. Near-real-time tracking of gaps in prevention of mother-to-child transmission of HIV in three districts of Kwa-Zulu Natal province, South Africa. *S Afr Med J*. 2018;**108**(4):319–24.

44. Mujugira A, Baeten J, Kidoguchi L, Haberer J, Celum C, Donnell D, et al. High levels of viral suppression among east African HIV-infected women and men in serodiscordant partnerships initiating antiretroviral therapy with high CD4 counts and during pregnancy. *AIDS Res Hum Retroviruses*. 2018;**34**(2):140–7.

45. North C, Allen J, Okello S, Sentongo R, Kakuhikire B, Ryan E, et al. HIV infection, pulmonary tuberculosis, and COPD in rural Uganda: A cross-sectional study. *Lung*. 2018;**196**(1):49–57.

46. Okawa S, Mwanza Kabaghe S, Mwiya M, Kikuchi K, Jimba M, Kankasa C, et al. Psychological well-being and adherence to antiretroviral therapy among adolescents living with HIV in Zambia. *AIDS Care*. 2018;**30**(5):634–42.

47. Ousley J, Niyibizi A, Wanjala S, Vandenbulcke A, Kirubi B, Omwoyo W, et al. High proportions of patients with advanced HIV are antiretroviral therapy experienced: hospitalization outcomes from 2 sub-Saharan African sites. *Clin Infect Dis*. 2018;**66**(Suppl 2):S126–32.

48. Seyoum M, Enawgaw B, Getaneh Z, Engidaye G, Asrie F, Melku M. Basic coagulation parameters among human immunodeficiency virus-infected adults in Gondar, northwest Ethiopia: a comparative cross-sectional study. *Biomed Res Int*. 2018;**Volume 201**.

49. Ugboaja J, Oguejiofor C, Ogelle O. Highly active antiretroviral therapy and cervical cytologic abnormalities among women with HIV infection in a limited-resource setting. *Int J Gynaecol Obs*. 2018;**140**(2):228–32.

50. Zhang Y, Fogel G, Guo X, Clarke W, Breaud A, Cummings V, et al. Antiretroviral drug use and HIV drug resistance among MSM and transgender women in sub-Saharan Africa. *AIDS*. 2018;**32**(10):1301–7.

51. Ayanga R, Namukwaya Z, Lugoloobi E, Mugerwa J, Afrika S, Kakande A, et al. Virological response among HIV-infected pregnant and lactating women initiated on option B+ attending the PMTCT program at Mulago national hospital, Kampala, Uganda. In: Proceedings of the 2015 International AIDS Conference. Vancouver, Canada; 2015.

52. Jain V, Chamie G, Amanyire G, Kwarisiim D, Kabami J, Chamie J, et al. Population-level HIV RNA and CD4+ distribution in a rural Ugandan community with widespread community HIV testing and universal ART access. In: Proceedings of the 2015 Conference on Retroviruses and Opportunistic Infections. Seattle, WA; 2015.

53. Rhein J. Detrimental outcomes of unmasking cryptococcal meningitis with recent ART initiation. *Open Forum Infect Dis*. 2018;**5**(8):ofy122.

54. Schwartz S. Safer conception delayed by lack of HIV viral suppression. In: Proceedings of the 2015 International AIDS Conference. Vancouver, Canada; 2015.

55. Aston S, Ho A, Jary H, Everett D, Mwandumba H, Heyderman R, et al. Aetiology and outcome of community-acquired pneumonia in HIV-infected Malawian adults. In: Proceedings of the 2016 Conference on Retroviruses and Opportunistic Infections. Boston, MA; 2016.

56. Casavant D, Honwana I, MacKellar R, Thompson R, Nelson J, Bonzela B, et al. Home-based HIV testing and new HIV diagnoses in Chókwè District, Mozambique. In: Proceedings of the 2016 Conference on Retroviruses and Opportunistic Infections. Boston, MA; 2016.

57. Kharsany A, Cawood C, Khanyile D, Grobler A, Puren A, Kufa-Chakeza T, et al. Strengthening HIV surveillance in the antiretroviral therapy era: Baseline findings of HIV prevalence and incidence from KwaZulu-Natal, South Africa. In: Proceedings of the 2016 International AIDS Conference. Durban, South Africa; 2016.

58. Lahuerta P, Bouyagui T, Patnaik N, Telly J, Knox T, Ballo A. HIV prevalence and risk factors in men who have sex with men in Bamako, Mali. In: Proceedings of the 2016 Conference on Retroviruses and Opportunistic Infections. Boston, MA; 2016.

59. MacLeod D, Tshepo W, Bor J, Fraser N, Shubber Z, Sanne I, et al. Measuring viral load suppression in South Africa using a novel, national database. In: Proceedings of the 2016 Conference on Retroviruses and Opportunistic Infections. Boston, MA; 2016.

60. ICAP at Columbia University. Malawi population-based HIV impact assessment (MPHIA). New York City; 2016.

61. ICAP at Columbia University. Tanzania HIV impact survey (THIS). New York City; 2016.

62. ICAP at Columbia University. Uganda population-based HIV impact assessment (UPHIA). New York City; 2016.

63. ICAP at Columbia University. Zambia population-based HIV impact assessment (ZAMPHIA). New York City; 2016.

64. ICAP at Columbia University. Zimbabwe population-based HIV impact assessment (ZIMPHIA). New York City; 2016.

65. Ribakare M, Nsanzimana M, Ndahimana J, Rusisiro B, Niyibizi G, Uwizihiwe J, et al. Feasibility of the third 90-90-90 target: Viral load coverage and outcomes in Rwanda. In: Proceedings of the 2016 Conference on Retroviruses and Opportunistic Infections. Boston, MA; 2016.

66. Tippett Barr B, Schouten E, van Oosterhout J, Gupta S, Phiri H, Thindwa D. National HIV transmission in 4-12 week olds in Malawi's PMTCT option B+ program. In: Proceedings of the 2016 Conference on Retroviruses and Opportunistic Infections. Boston, MA; 2016.

67. Abuogi L, Akama E, Nimz A, Blat C, Cohen C, Oyaro P. Retention and viral suppression of newly diagnosed and known HIV-positive pregnant women on option B+ in Kenya. In: Proceedings of the 2017 International AIDS Conference. Paris, France; 2017.

68. Breton G, Ndawinz J, Nimbona P, O'Sylla J, Akamba M, Mbangue S, et al. HIV viral load monitoring in four west and central African countries: How is virological failure managed by caregivers in the OPP-ERA project? In: Proceedings of the 2017 International AIDS Conference. Paris, France; 2017.

69. Couderc C, Dah T, Diallo F, Kouamé M, Agboyibor R, Bernier A, et al. Feasibility and acceptability of immediate ART initiation in MSM in west Africa (CohMSM ANRS 12324 - Expertise France). In: Proceedings of the 2017 International AIDS Conference. Paris, France; 2017.

70. Kharsany A, Cawood C, Khanyile D, Grobler A, Lewis L, Puren A. Population HIV viral load metrics: Findings from household surveys in KwaZulu-Natal, South Africa. In: Proceedings of the 2017 International AIDS Conference. Paris, France; 2017.

71. Kiene S, Naigino R, Sileo K, Kalichman S, Menzies N, Bategenya M, et al. Only 36% of adults living with HIV aware of their HIV+ status but progress in ART coverage and viral suppression in a subnational survey in Uganda: Progress towards UNAIDS 90-90-90. In: Proceedings of the 2017 International AIDS Conference. Paris, France; 2017.

72. Malaba T, Newell M, Madlala H, Perez A, Gray C, Myer L. Methods of gestational age (GA) assessment influence the observed association between ART exposure and preterm delivery (PTD): A prospective study in Cape Town, South Africa. In: Proceedings of the

2017 International AIDS Conference. Paris, France; 2017.

73. Mendelsohn S, Aluda C, Ortuno R, Shigayeva A, Hilderband K, Goemaere E. HIV and tuberculosis in Malawian prisons: A comprehensive prevention, screening and management programme. In: Proceedings of the 2017 International AIDS Conference. Paris, France; 2017.

74. Mutenda N, Taffa N, Baughman D, Agovi A, De Klerk M, Sawadogo S, et al. Outcomes of routine viral load monitoring in Namibia's national antiretroviral treatment program. In: Proceedings of the 2017 International AIDS Conference. Paris, France; 2017.

75. Ndagire H, Kazibwe F, Ajok F, Mugume A. The role of peer-to-peer model in achieving 90-90-90 among female sex workers in east central Uganda. In: Proceedings of the 2017 International AIDS Conference. Paris, France; 2017.

76. Nicholas S, Schramm B, Poulet E, Ajule E, Candiru H, Adroa P, et al. Viral load monitoring with SAMBA-1, a semi-quantitative nearly point-of-care method in Arua, a rural district in Uganda. In: Proceedings of the 2017 International AIDS Conference. Paris, France; 2017.

77. Nikuze A, Wanjala S, Ben-Farhat J, Omwoyo W, O'Alliance L. HIV incidence, cascade, and testing among mothers in western Kenya. In: Proceedings of the 2017 Conference on Retroviruses and Opportunistic infections. Seattle; 2017.

78. Nsumba M, Zimaze R, Kaimal A, Bakabikoba H, Mubiru F, Lamorde M, et al. Risk factors for viral failure among HIV patients on antiretroviral therapy (ART) at the Infectious Diseases Institute Kampala Uganda. In: Proceedings of the 2017 International AIDS Conference. Paris, France; 2017.

79. Olang'o P, Mwimali P, Karuga R, Seii M. Tit integration of HIV testing and prevention services among men who have sex with men (MSM) in the government owned health facilities in western Kenya. In: Proceedings of the 2017 International AIDS Conference. Paris, France; 2017.

80. Pathmanathan I, Pasipamire M, Pals S, Dokubo E, Preko P, Ao T, et al. High uptake of antiretroviral therapy among HIV-positive TB patients receiving co-located services in Swaziland. In: Proceedings of the 2017 International AIDS Conference. Paris, France; 2017.

81. ICAP at Columbia University. Lesotho population-based HIV impact assessment (LePHIA). New York City; 2017.

82. ICAP at Columbia University. Swaziland HIV incidence measurement survey 2 - A population-based HIV impact assessment (SHIMS2). New York City; 2017.

83. Dadabhai S, Gadama L, Chamanga R, Kawalazira R, Katumbi C, Dula D, et al. Pregnancy outcomes in the era of universal HAART in Africa (The POISE Study). In: Proceedings of the 2018 Conference on Retroviruses and Opportunistic Infections. Boston, MA; 2018.

84. Hoffmann C, Milovanovic M, Kerrigan D, Martinson N, Variava E. Post-hospital mortality and readmission among HIV-infected adults in South Africa. In: Proceedings of the 2018 Conference on Retroviruses and Opportunistic Infections. Boston, MA; 2018.

85. Human Sciences Research Council. The fifth South African national HIV prevalence, incidence, behaviour and communication survey, 2017 (SABSSM V). Pretoria, South Africa; 2017.

86. Matthews L, Orrell C, Bwana B, Asiimwe S, Amanyire G, Musinguzi N, et al. ART adherence among pregnant and non-pregnant women in S. Africa and Uganda. In: Proceedings of the 2018 Conference on Retroviruses and Opportunistic Infections. Boston, MA; 2018.

87. ICAP at Columbia University. Cameroon HIV impact assessment (CAMPHIA). 2018.

88. ICAP at Columbia University. Cote d'Ivoire HIV impact assessment (CIPHIA). 2018.

89. ICAP at Columbia University. Namibia HIV impact assessment (NAMPHIA). 2018.

90. ICAP at Columbia University. Ethiopia HIV impact assessment (EPHIA). 2018.

91. Price J, Vwalika B, Winston J, Kasaro M, Mwape H, Chi B, et al. Preconceptional ART and spontaneous preterm birth in an urban Zambian cohort. In: Proceedings of the 2018 Conference on Retroviruses and Opportunistic Infections. Boston, MA; 2018.

92. Zielinski-Gutirrez E, Odongo S, Kwaro D, Mutai K. Fisherfolk at higher risk than their neighbors: findings from western Kenya. In: Proceedings of the 2018 Conference on Retroviruses and Opportunistic Infections. Boston, MA; 2018.
