# Supplementary material for: Evidence of sociodemographic heterogeneity across the HIV treatment cascade and progress towards 90‐90‐90 in sub‐Saharan Africa – a systematic review and meta‐analysis
Source: J Int AIDS Soc. 2020 Mar 9;23(3):e25470. doi: 10.1002/jia2.25470 (PMC7062634; doi:10.1002/jia2.25470)
Supplement: Supplementary file 3 — File S3. Risk of bias assessment results by study and in aggregate. [file JIA2-23-e25470-s003.pdf]

Additional File 3: Risk of Bias Assessment

Below are summary and detailed findings of the risk of bias assessment for included peer-reviewed articles as well as population-based surveys.

Figure 1: Overall and Domain-Specific Distribution of Risk of Bias for Included Peer-Reviewed and Population-Based Surveys

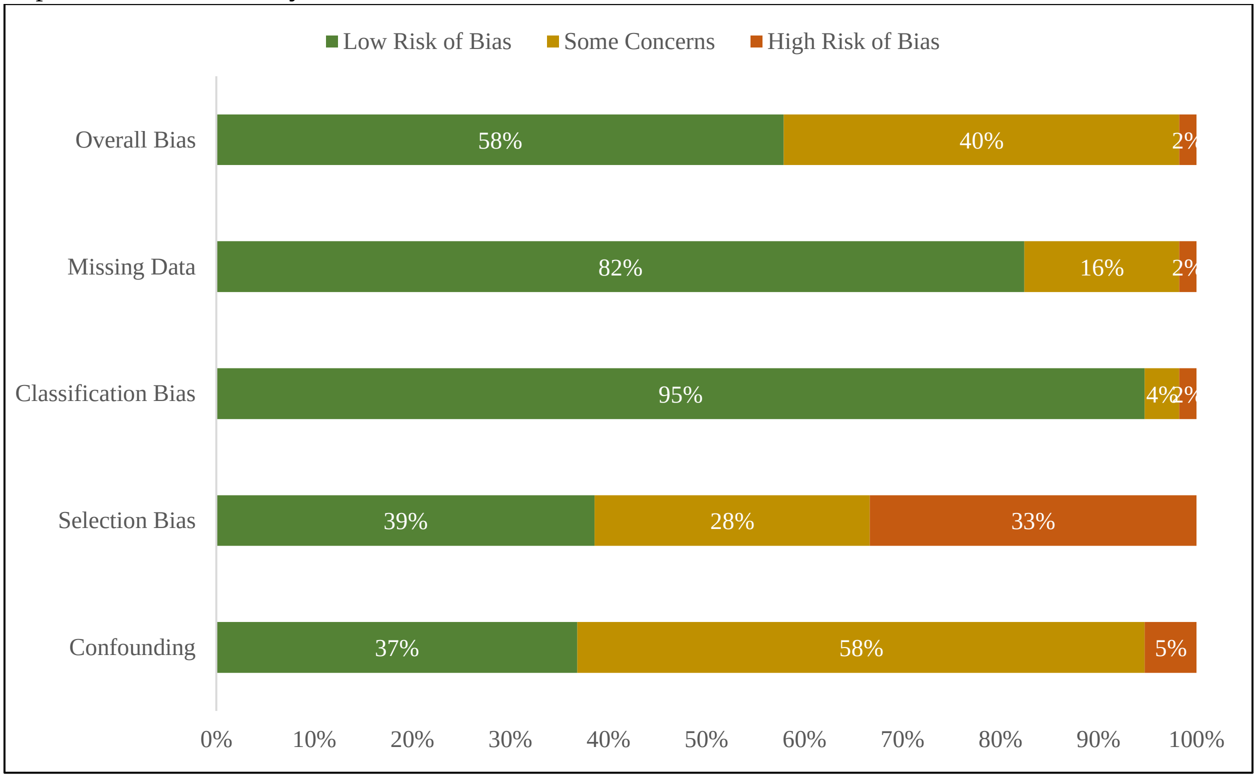

Table 1: Detailed Risk of Bias Assessment Findings by Domain

Author	Year	Confounding	Selection Bias	Classification Bias	Missing Data	Overall Bias
Ahmed	2017	Some Concerns	High Risk of Bias	Low Risk of Bias	Low Risk of Bias	Some Concerns
Asiimwe	2017	Some Concerns	Low Risk of Bias	Low Risk of Bias	Low Risk of Bias	Low Risk of Bias
Baeten	2016	Some Concerns	High Risk of Bias	Low Risk of Bias	Low Risk of Bias	Some Concerns
Barnabas	2016	Some Concerns	High Risk of Bias	Low Risk of Bias	Low Risk of Bias	Some Concerns
Boyer	2016	Some Concerns	High Risk of Bias	Low Risk of Bias	Low Risk of Bias	Some Concerns
Brown	2018	Low Risk of Bias	Low Risk of Bias	Low Risk of Bias	Low Risk of Bias	Low Risk of Bias
Chanda	2017	Some Concerns	High Risk of Bias	Low Risk of Bias	Low Risk of Bias	Some Concerns
Charurat	2015	Some Concerns	High Risk of Bias	Low Risk of Bias	Some Concerns	Some Concerns
Collins	2016	Low Risk of Bias	High Risk of Bias	Low Risk of Bias	Low Risk of Bias	Low Risk of Bias
Elul	2017	Some Concerns	Some Concerns	Low Risk of Bias	Some Concerns	Some Concerns
Flynn	2017	Some Concerns	High Risk of Bias	Low Risk of Bias	Low Risk of Bias	Some Concerns
Gaolathe	2016	Low Risk of Bias	Low Risk of Bias	Low Risk of Bias	Low Risk of Bias	Low Risk of Bias
Grabowski	2017	Low Risk of Bias	Low Risk of Bias	Low Risk of Bias	Low Risk of Bias	Low Risk of Bias
Grobler	2018	Low Risk of Bias	Low Risk of Bias	Low Risk of Bias	Low Risk of Bias	Low Risk of Bias
Hayes	2017	Low Risk of Bias	Low Risk of Bias	Low Risk of Bias	Low Risk of Bias	Low Risk of Bias
Hoffman	2017	Some Concerns	Some Concerns	Low Risk of Bias	Low Risk of Bias	Low Risk of Bias
Holmes	2018	Some Concerns	Low Risk of Bias	Low Risk of Bias	Low Risk of Bias	Low Risk of Bias
Kerrigan	2017	Some Concerns	High Risk of Bias	Some Concerns	Low Risk of Bias	Some Concerns
Laher	2018	High Risk of Bias	High Risk of Bias	Low Risk of Bias	Low Risk of Bias	Some Concerns
Lancaster	2016	Some Concerns	High Risk of Bias	High Risk of Bias	Low Risk of Bias	High Risk of Bias
Lyons	2017	Some Concerns	High Risk of Bias	Some Concerns	Low Risk of Bias	Some Concerns
Mafigiri	2017	Some Concerns	Some Concerns	Low Risk of Bias	Low Risk of Bias	Low Risk of Bias
Mujugira	2018	Some Concerns	Some Concerns	Low Risk of Bias	Low Risk of Bias	Low Risk of Bias
Myburgh	2017	High Risk of Bias	Some Concerns	Low Risk of Bias	Low Risk of Bias	Some Concerns
Myer	2015	Some Concerns	Some Concerns	Low Risk of Bias	Low Risk of Bias	Low Risk of Bias
North	2018	Some Concerns	High Risk of Bias	Low Risk of Bias	Low Risk of Bias	Some Concerns
Ortblad	2017	Some Concerns	High Risk of Bias	Low Risk of Bias	Some Concerns	Some Concerns
Petersen	2017	Low Risk of Bias	Low Risk of Bias	Low Risk of Bias	Low Risk of Bias	Low Risk of Bias
Phiri	2017	Some Concerns	Some Concerns	Low Risk of Bias	Some Concerns	Some Concerns
Rao	2016	High Risk of Bias	High Risk of Bias	Low Risk of Bias	Low Risk of Bias	Some Concerns
Rosenberg	2015	Some Concerns	Some Concerns	Low Risk of Bias	Low Risk of Bias	Low Risk of Bias
Rosenberg	2017	Some Concerns	Some Concerns	Low Risk of Bias	Low Risk of Bias	Low Risk of Bias
Ruria	2017	Some Concerns	Some Concerns	Low Risk of Bias	Low Risk of Bias	Low Risk of Bias
Ruzagira	2017	Low Risk of Bias	Low Risk of Bias	Low Risk of Bias	Some Concerns	Low Risk of Bias
Schwartz	2017	Some Concerns	High Risk of Bias	Low Risk of Bias	Low Risk of Bias	Some Concerns
Stahlman	2016	Some Concerns	High Risk of Bias	Low Risk of Bias	Some Concerns	Some Concerns
Ugbaoja	2018	Some Concerns	Some Concerns	Low Risk of Bias	Low Risk of Bias	Low Risk of Bias
Okawa	2018	Some Concerns	Some Concerns	Low Risk of Bias	Low Risk of Bias	Low Risk of Bias
Chagomerana	2018	Low Risk of Bias	Some Concerns	Low Risk of Bias	Some Concerns	Low Risk of Bias
Moyo	2018	Some Concerns	Some Concerns	Low Risk of Bias	Some Concerns	Some Concerns
Ousley	2018	Some Concerns	Some Concerns	Low Risk of Bias	Some Concerns	Some Concerns
Hansoti	2018	Some Concerns	Some Concerns	Low Risk of Bias	High Risk of Bias	Some Concerns
Seyoum	2018	Some Concerns	Low Risk of Bias	Low Risk of Bias	Low Risk of Bias	Low Risk of Bias
Zhang	2018	Some Concerns	High Risk of Bias	Low Risk of Bias	Low Risk of Bias	Some Concerns
Ephraim	2018	Some Concerns	High Risk of Bias	Low Risk of Bias	Low Risk of Bias	Some Concerns
Population-Based Surveys						
SABSSMV	2017	Low Risk of Bias	Low Risk of Bias	Low Risk of Bias	Low Risk of Bias	Low Risk of Bias
Cameroon PHIA	2018	Low Risk of Bias	Low Risk of Bias	Low Risk of Bias	Low Risk of Bias	Low Risk of Bias
Cote d'Ivoire PHIA	2018	Low Risk of Bias	Low Risk of Bias	Low Risk of Bias	Low Risk of Bias	Low Risk of Bias
Nambia PHIA	2018	Low Risk of Bias	Low Risk of Bias	Low Risk of Bias	Low Risk of Bias	Low Risk of Bias
Ethiopia PHIA	2018	Low Risk of Bias	Low Risk of Bias	Low Risk of Bias	Low Risk of Bias	Low Risk of Bias
Lesotho PHIA	2017	Low Risk of Bias	Low Risk of Bias	Low Risk of Bias	Low Risk of Bias	Low Risk of Bias
Malawi PHIA	2016	Low Risk of Bias	Low Risk of Bias	Low Risk of Bias	Low Risk of Bias	Low Risk of Bias
Swaziland PHIA	2017	Low Risk of Bias	Low Risk of Bias	Low Risk of Bias	Low Risk of Bias	Low Risk of Bias
Tanzania PHIA	2017	Low Risk of Bias	Low Risk of Bias	Low Risk of Bias	Low Risk of Bias	Low Risk of Bias
Uganda PHIA	2017	Low Risk of Bias	Low Risk of Bias	Low Risk of Bias	Low Risk of Bias	Low Risk of Bias
Zambia PHIA	2016	Low Risk of Bias	Low Risk of Bias	Low Risk of Bias	Low Risk of Bias	Low Risk of Bias
Zimbabwe PHIA	2016	Low Risk of Bias	Low Risk of Bias	Low Risk of Bias	Low Risk of Bias	Low Risk of Bias
